# Supplementary material for: Irrigated areas drive irrigation water withdrawals
Source: Nat Commun. 2021 Jul 26;12:4525. doi: 10.1038/s41467-021-24508-8 (PMC8313559; doi:10.1038/s41467-021-24508-8)
Supplement: Supplementary file 1 — Supplementary Material [file 41467_2021_24508_MOESM1_ESM.pdf]

Irrigated areas drive irrigation water withdrawals

Supplementary Materials

Arnald Puy*[1,2], Emanuele Borgonovo[3], Samuele Lo Piano[4], Simon A. Levin[1], and Andrea Saltelli[5]

[1] *Department of Ecology and Evolutionary Biology, M31 Guyot Hall, Princeton University, New Jersey 08544, USA. E-Mail: apuy@princeton.edu*
[2] *Centre for the Study of the Sciences and the Humanities (SVT), University of Bergen, Parkveien 9, PB 7805, 5020 Bergen, Norway.*
[3] *Department of Decision Sciences and BIDSA, Boconi University, Via Roentgen 1, 20136 Milan, Italy*
[4] *University of Reading, School of the Built Environment, JJ Thompson Building, Whiteknights Campus, Reading, RG6 6AF, United Kingdom*
[5] *Open Evidence Research, Universitat Oberta de Catalunya (UOC), Barcelona, Spain.*

Contents

1 Table **2**

2 Figures **4**

*Corresponding author

1 Table

Table S1: Countries missing in each data set.

	Country	Water Dataset
1	Saint Lucia	Liu et al. 2016
2	Swaziland	Liu et al. 2016
3	Belize	Aquastat
4	Bosnia and Herzegovina	Aquastat
5	Cape Verde	Aquastat
6	Croatia	Aquastat
7	Cuba	Aquastat
8	El Salvador	Aquastat
9	Grenada	Aquastat
10	Guyana	Aquastat
11	Haiti	Aquastat
12	Ireland	Aquastat
13	Lebanon	Aquastat
14	Luxembourg	Aquastat
15	Malaysia	Aquastat
16	Panama	Aquastat
17	Peru	Aquastat
18	Saint Lucia	Aquastat
19	Sudan	Aquastat
20	Suriname	Aquastat
21	Swaziland	Aquastat
22	Trinidad and Tobago	Aquastat
23	Uruguay	Aquastat
24	Grenada	LPJmL
25	Malta	LPJmL
26	Saint Lucia	LPJmL
27	Seychelles	LPJmL
28	Swaziland	LPJmL
29	Argentina	PCR-GLOBWB
30	Canada	PCR-GLOBWB
31	Grenada	PCR-GLOBWB
32	Malta	PCR-GLOBWB
33	Portugal	PCR-GLOBWB
34	Russia	PCR-GLOBWB
35	Saint Lucia	PCR-GLOBWB
36	Seychelles	PCR-GLOBWB
37	Swaziland	PCR-GLOBWB
38	Grenada	H08
39	Malta	H08

40	Saint Lucia	H08
41	Seychelles	H08
42	Swaziland	H08
43	Grenada	WaterGap
44	Malta	WaterGap
45	Saint Lucia	WaterGap
46	Seychelles	WaterGap
47	Swaziland	WaterGap
48	Grenada	DBHM
49	Malta	DBHM
50	Mauritius	DBHM
51	Saint Lucia	DBHM
52	Seychelles	DBHM
53	Swaziland	DBHM
54	Grenada	MPI-HM
55	Malta	MPI-HM
56	Saint Lucia	MPI-HM
57	Seychelles	MPI-HM
58	Swaziland	MPI-HM
59	Grenada	VIC
60	Malta	VIC
61	Saint Lucia	VIC
62	Seychelles	VIC
63	Swaziland	VIC
64	Cape Verde	CLM45
65	Grenada	CLM45
66	Malta	CLM45
67	Saint Lucia	CLM45
68	Seychelles	CLM45
69	Swaziland	CLM45

2 Figures

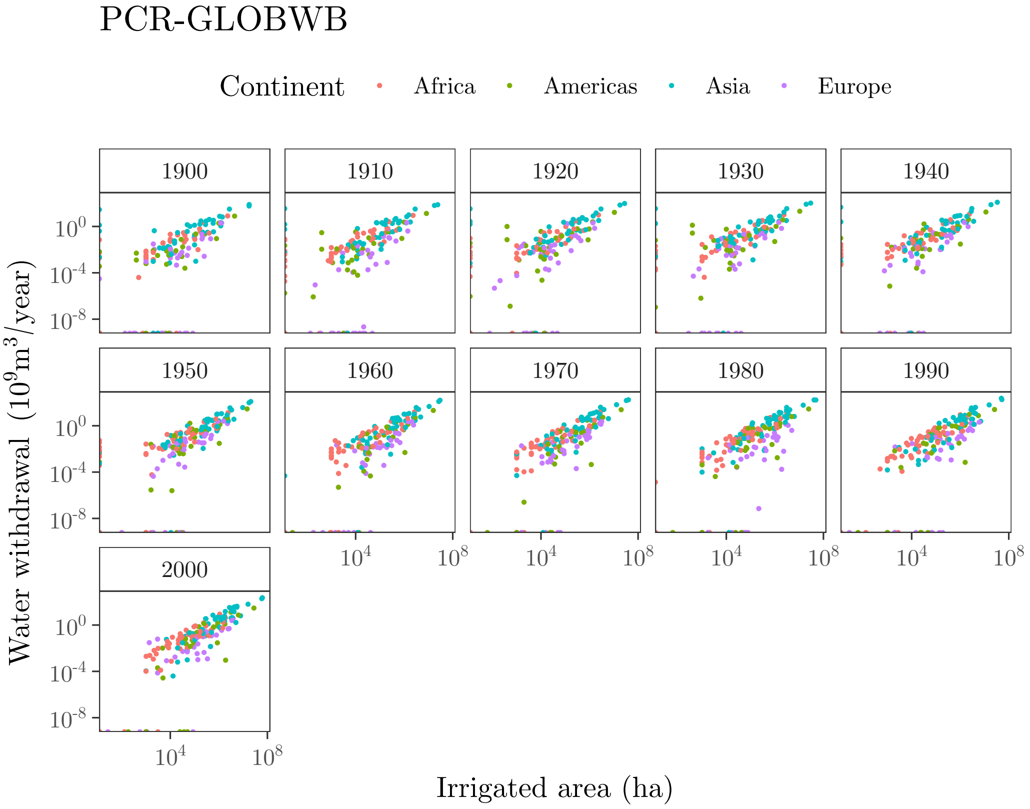

Figure S1: Historical relation between irrigated areas and irrigation water withdrawals (1900-2000). Data on historical irrigated areas was retrieved from the Historical Irrigation Data set (HID) [1], which follows the FAO-GMIA methodology. Data on historical irrigation water withdrawals was retrieved from ISIMIP [2]. Each dot is a country.

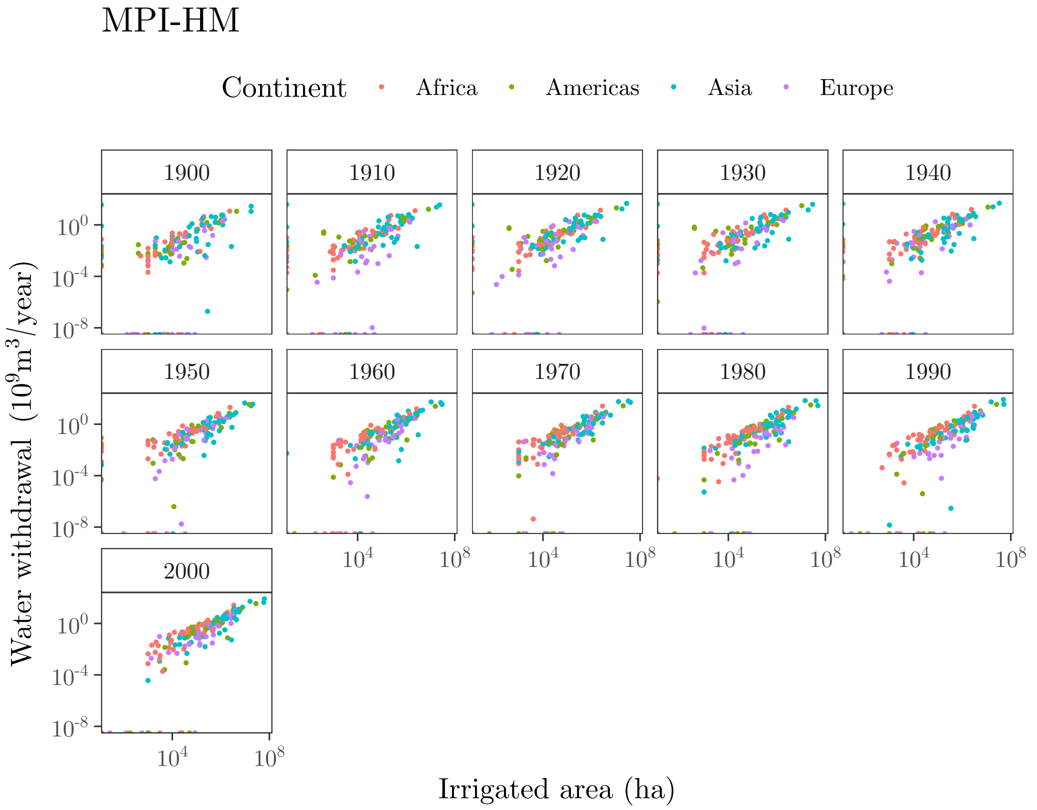

Figure S2: Historical relation between irrigated areas and irrigation water withdrawals (1900-2000). Data on historical irrigated areas was retrieved from the Historical Irrigation Data set (HID) [1], which follows the FAO-GMIA methodology. Data on historical irrigation water withdrawals was retrieved from ISIMIP [2]. Each dot is a country.

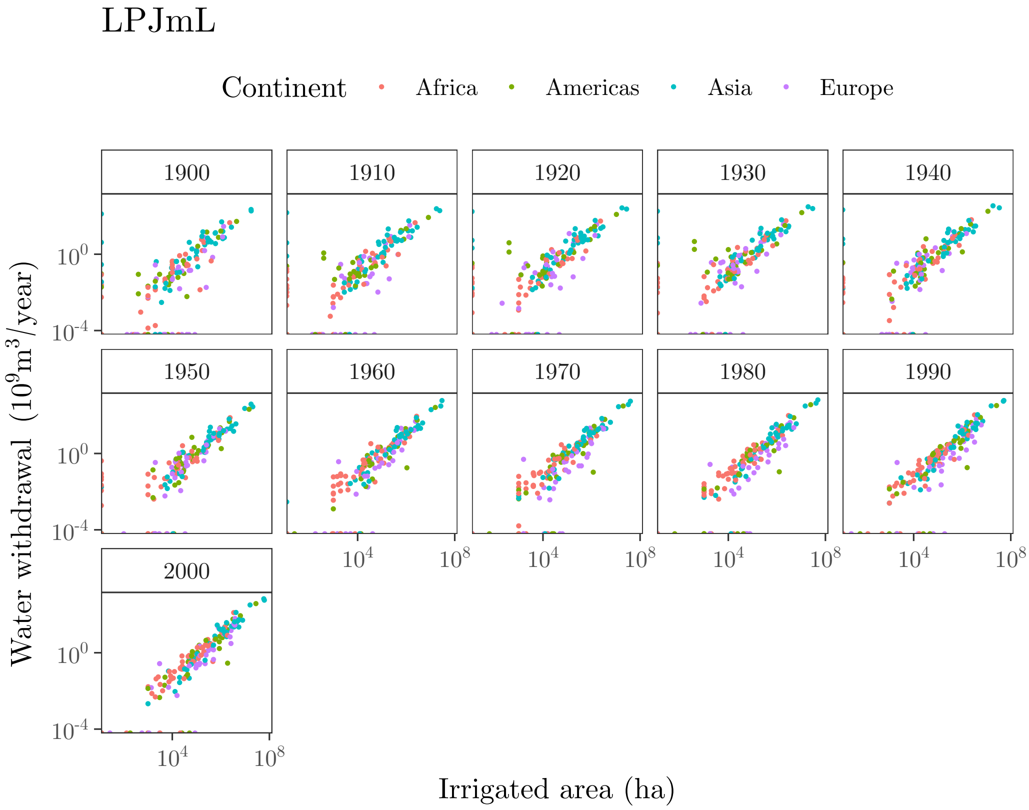

Figure S3: Historical relation between irrigated areas and irrigation water withdrawals (1900-2000). Data on historical irrigated areas was retrieved from the Historical Irrigation Data set (HID) [1], which follows the FAO-GMIA methodology. Data on historical irrigation water withdrawals was retrieved from ISIMIP [2]. Each dot is a country.

H08

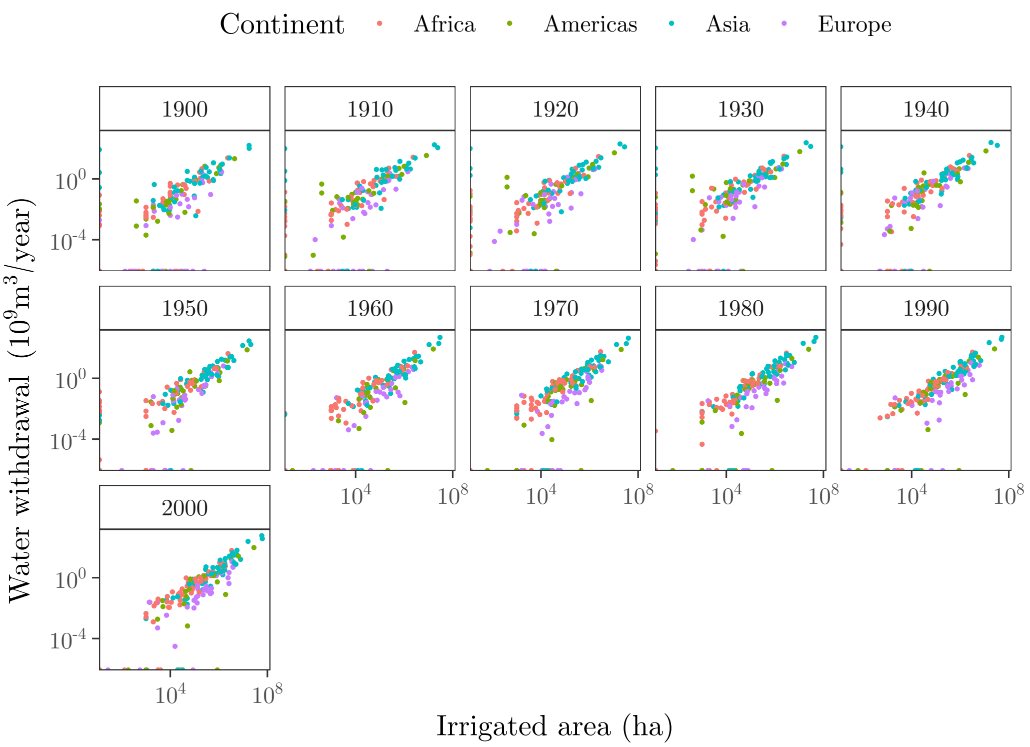

Figure S4: Historical relation between irrigated areas and irrigation water withdrawals (1900-2000). Data on historical irrigated areas was retrieved from the Historical Irrigation Data set (HID) [1], which follows the FAO-GMIA methodology. Data on historical irrigation water withdrawals was retrieved from ISIMIP [2]. Each dot is a country.

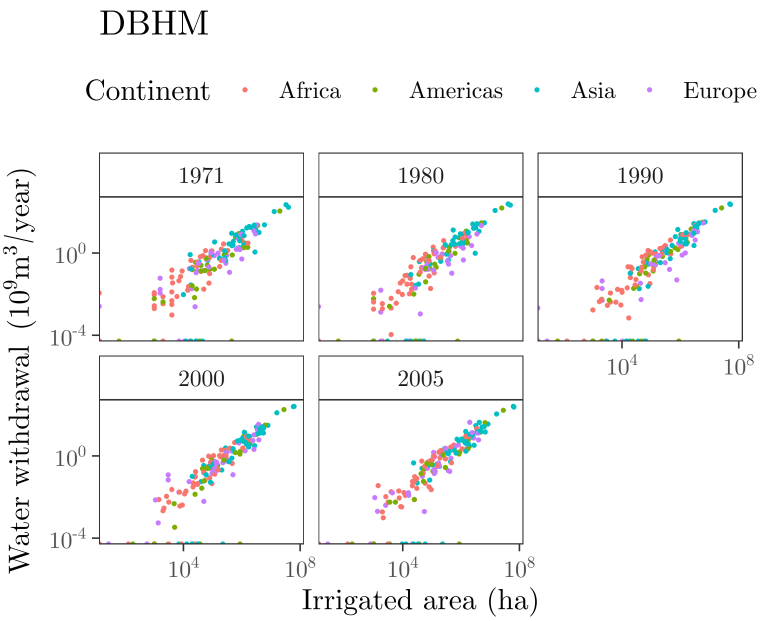

Figure S5: Historical relation between irrigated areas and irrigation water withdrawals (1971-2000). Data on historical irrigated areas was retrieved from the Historical Irrigation Data set (HID) [1], which follows the FAO-GMIA methodology. Data on historical irrigation water withdrawals was retrieved from ISIMIP [2]. Each dot is a country.

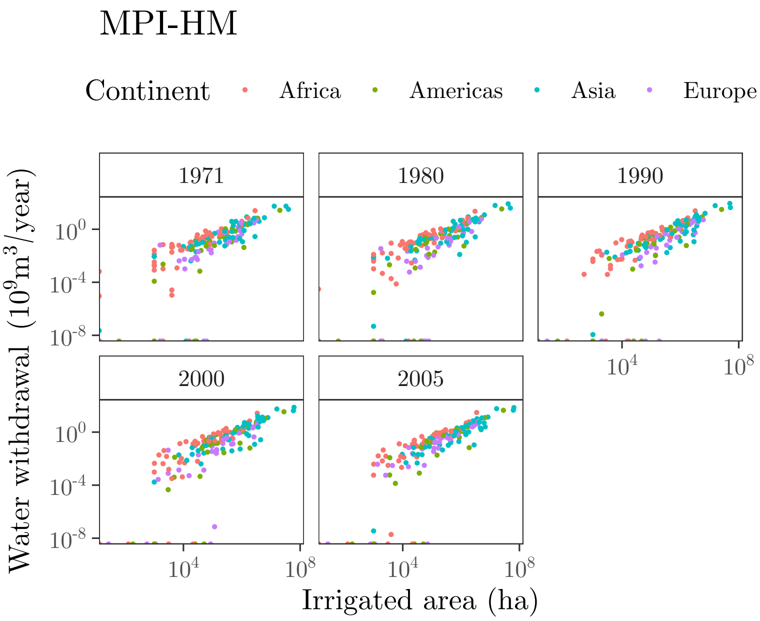

Figure S6: Historical relation between irrigated areas and irrigation water withdrawals (1971-2000). Data on historical irrigated areas was retrieved from the Historical Irrigation Data set (HID) [1], which follows the FAO-GMIA methodology. Data on historical irrigation water withdrawals was retrieved from ISIMIP [2]. Each dot is a country.

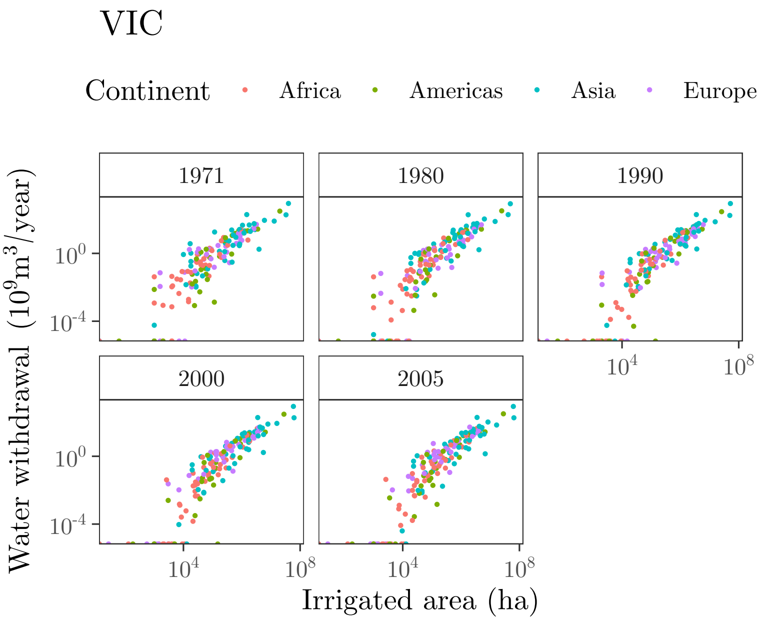

Figure S7: Historical relation between irrigated areas and irrigation water withdrawals (1971-2000). Data on historical irrigated areas was retrieved from the Historical Irrigation Data set (HID) [1], which follows the FAO-GMIA methodology. Data on historical irrigation water withdrawals was retrieved from ISIMIP [2]. Each dot is a country.

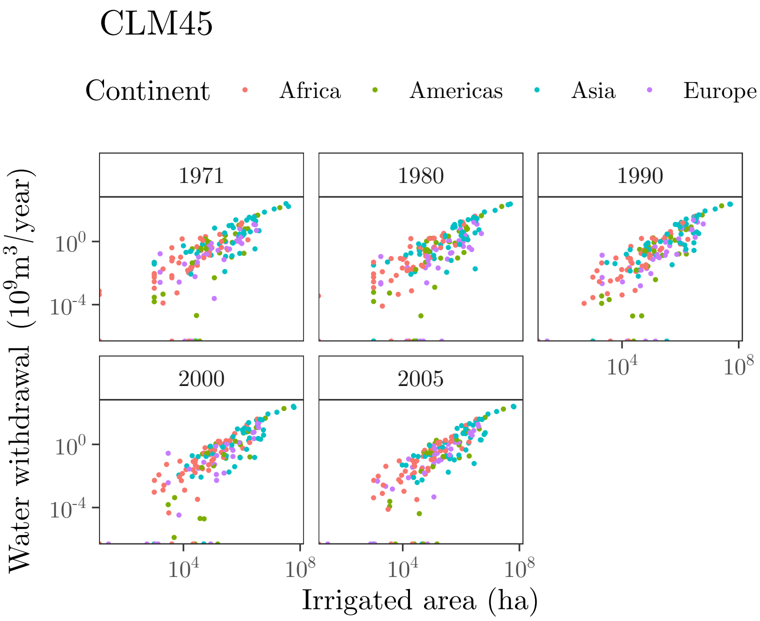

Figure S8: Historical relation between irrigated areas and irrigation water withdrawals (1971-2000). Data on historical irrigated areas was retrieved from the Historical Irrigation Data set (HID) [1], which follows the FAO-GMIA methodology. Data on historical irrigation water withdrawals was retrieved from ISIMIP [2]. Each dot is a country.

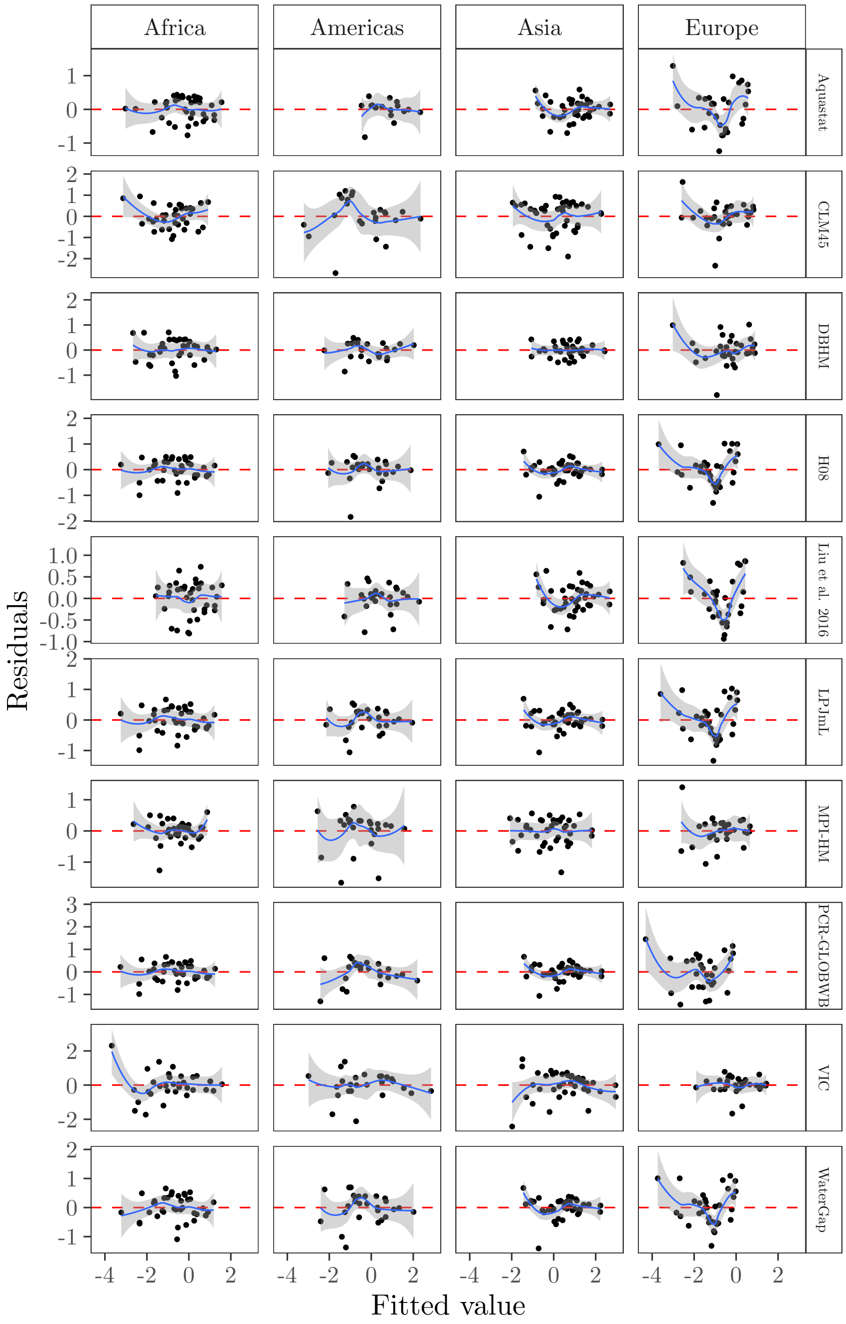

Figure S9: Residuals vs. fitted plot. A linear relationship between irrigated areas and irrigation water withdrawals is apparent for most combinations of continent and dataset except for Europe, which shows evident non-linearities in the case of Aquastat, Liu et al. 2016, LPJmL or WaterGap.

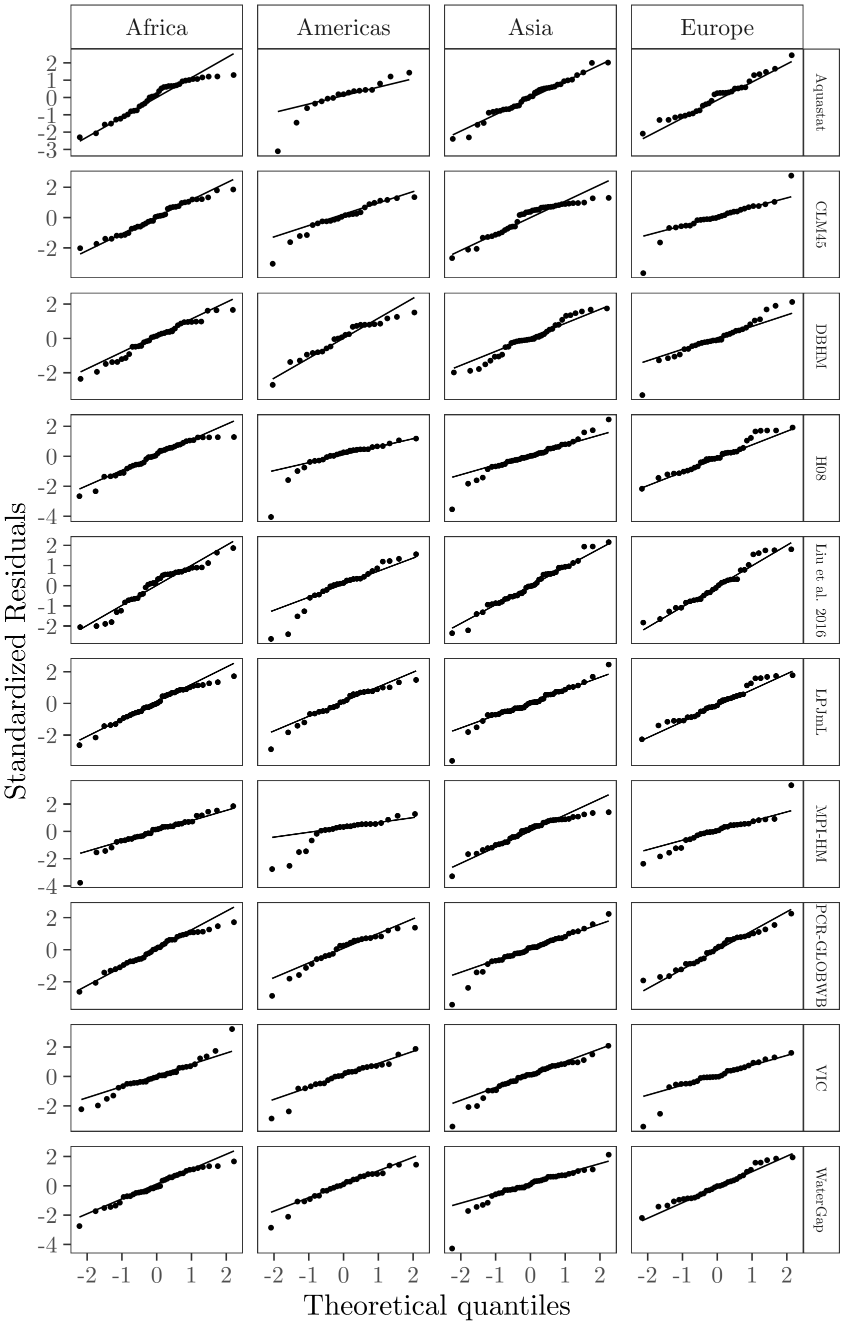

Figure S10: Normal Q-Q plot.

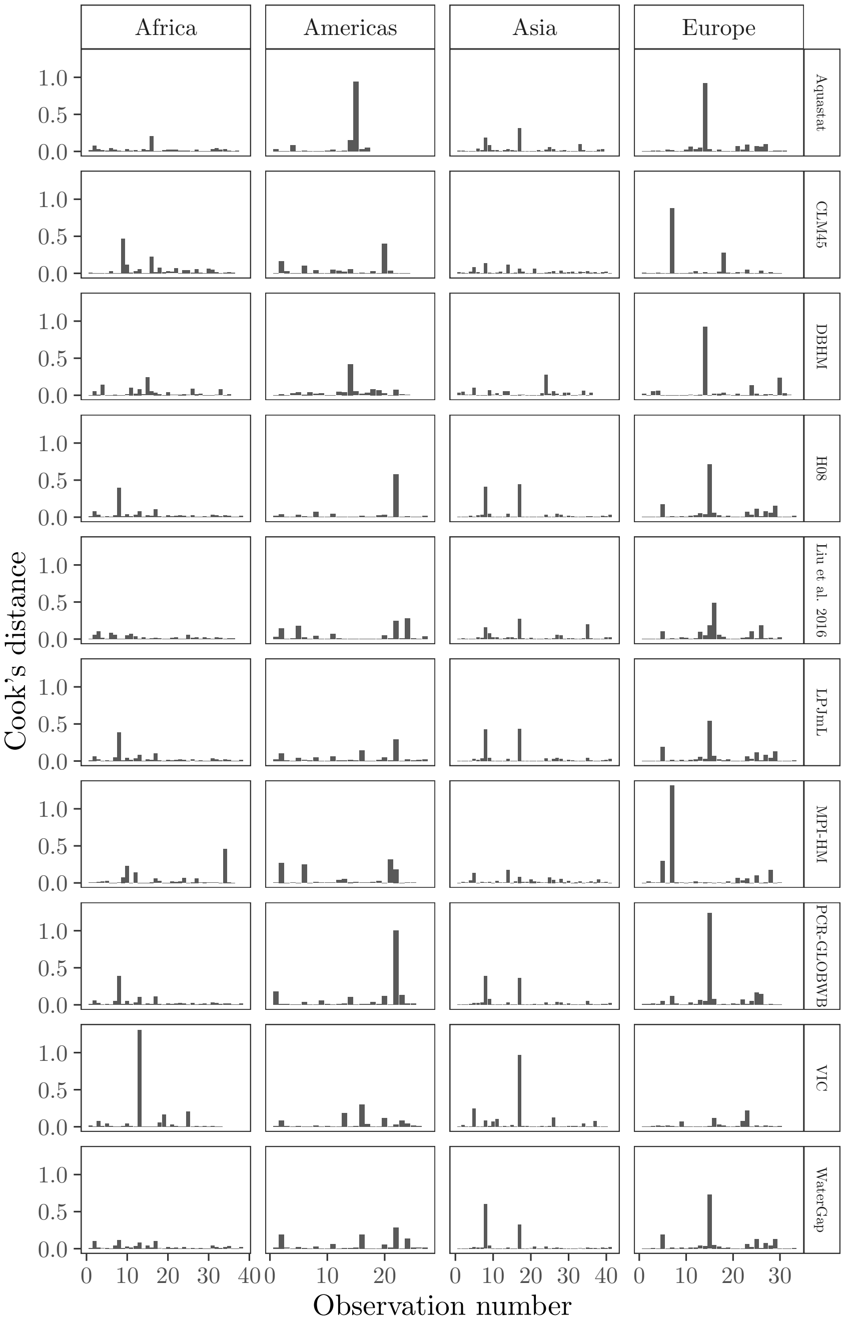

Figure S11: Cook's distance plot. Influential values are those substantially larger than the rest.

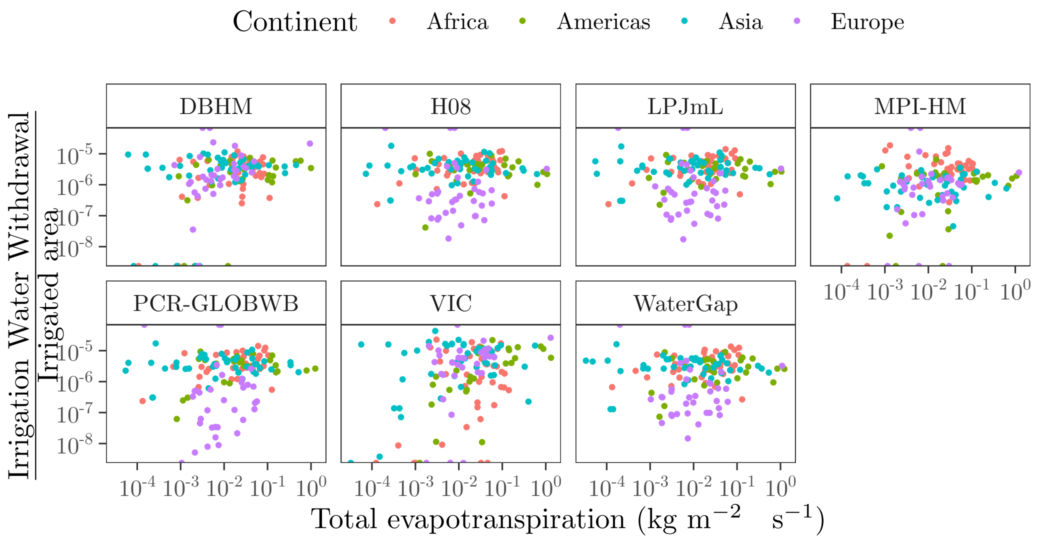

Figure S12: Scatterplots of (irrigation water withdrawal / irrigated areas) against total evapotranspiration. The data for total evapotranspiration has been retrieved from the Inter-Sectoral Impact Model Intercomparison Project (ISI-MIP) [2]. Only the GM that use total evapotranspiration in their calculations are plotted.

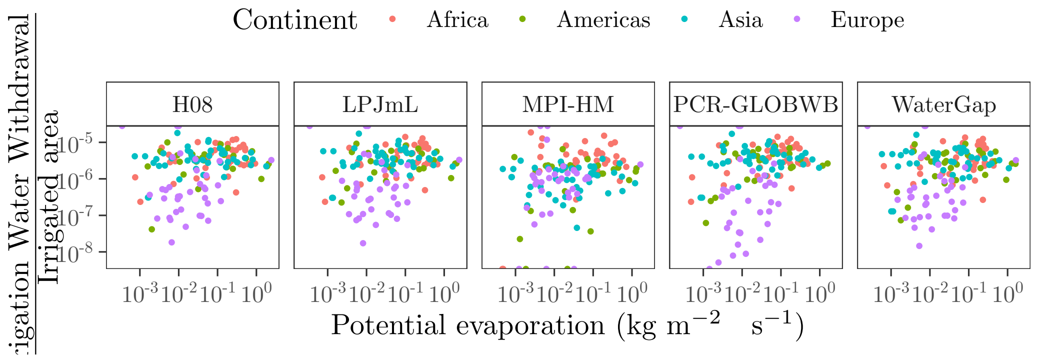

Figure S13: Scatterplots of (irrigation water withdrawal / irrigated areas) against potential evaporation. The data for potential evaporation has been retrieved from the Inter-Sectoral Impact Model Inter-comparison Project (ISI-MIP) [2]. Only the GM that use potential evaporation in their calculations are plotted.

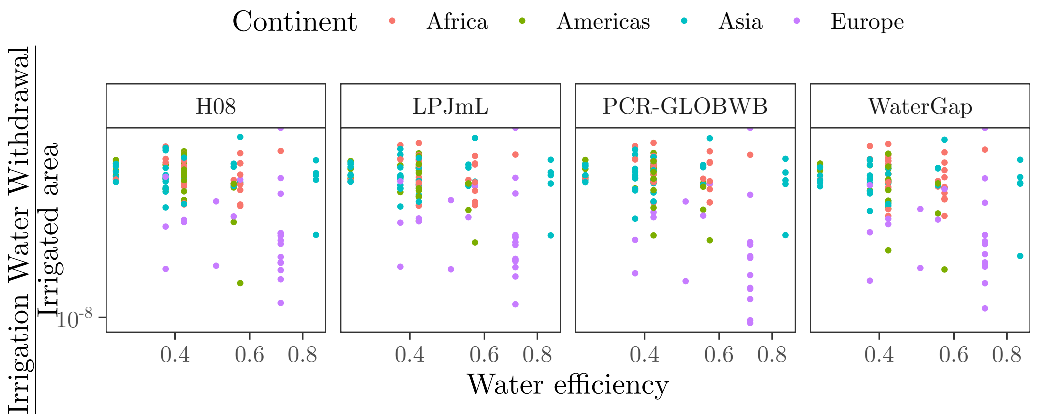

Figure S14: Scatterplots of (irrigation water withdrawal / irrigated area) against water efficiency. The values for water efficiency at the country level have been retrieved from Rohwer et al. [3]. Only those GM that rely on water efficiency for their calculations of irrigation water withdrawals are shown in the plot (see Wada et al. [4, Table S3]).

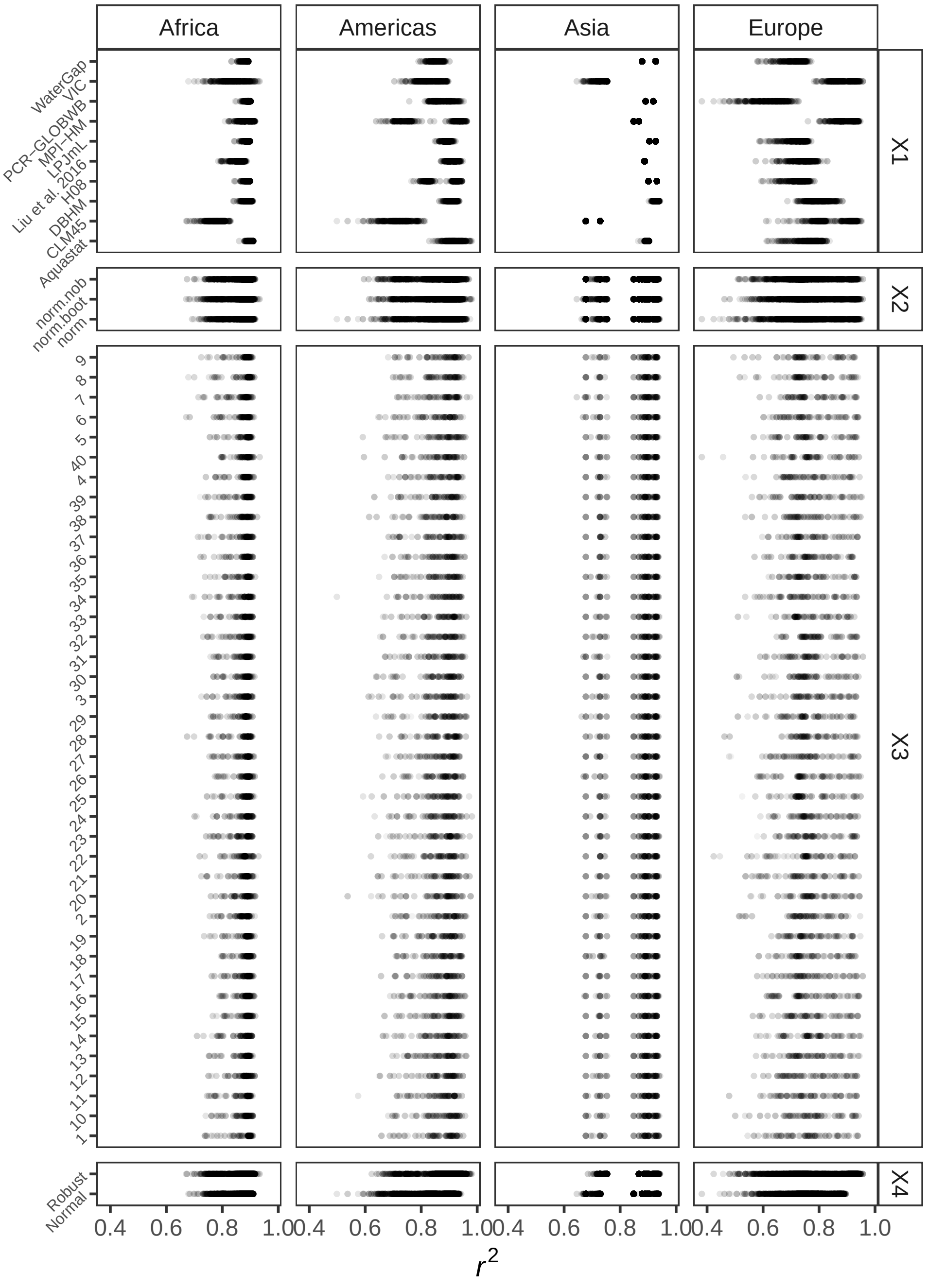

Figure S15: Scatterplots of model input versus model output.

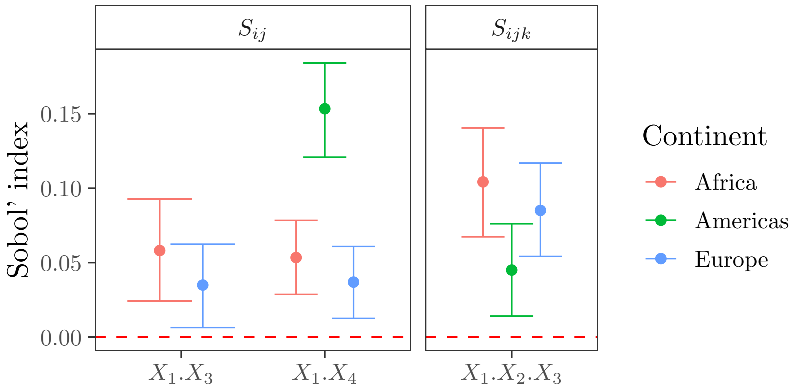

Figure S16: Second (S_{ij}) and third (S_{ijk}) -order Sobol' indices. The error bars show the 95% confidence interval after bootstrapping 500 times using the normal method. Only the effects whose lower bound does not overlap with zero are shown in the plot. The red, dashed horizontal line is at zero and marks the threshold below which S_{ij} and S_{ijk} are considered non-influential.

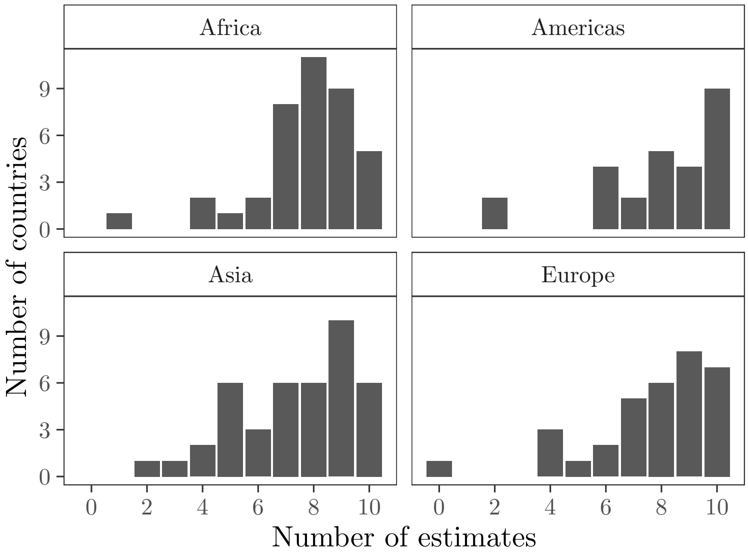

Figure S17: Number of countries with $0, 1, 2, ..., 10$ estimates bounded by our range of predictions.

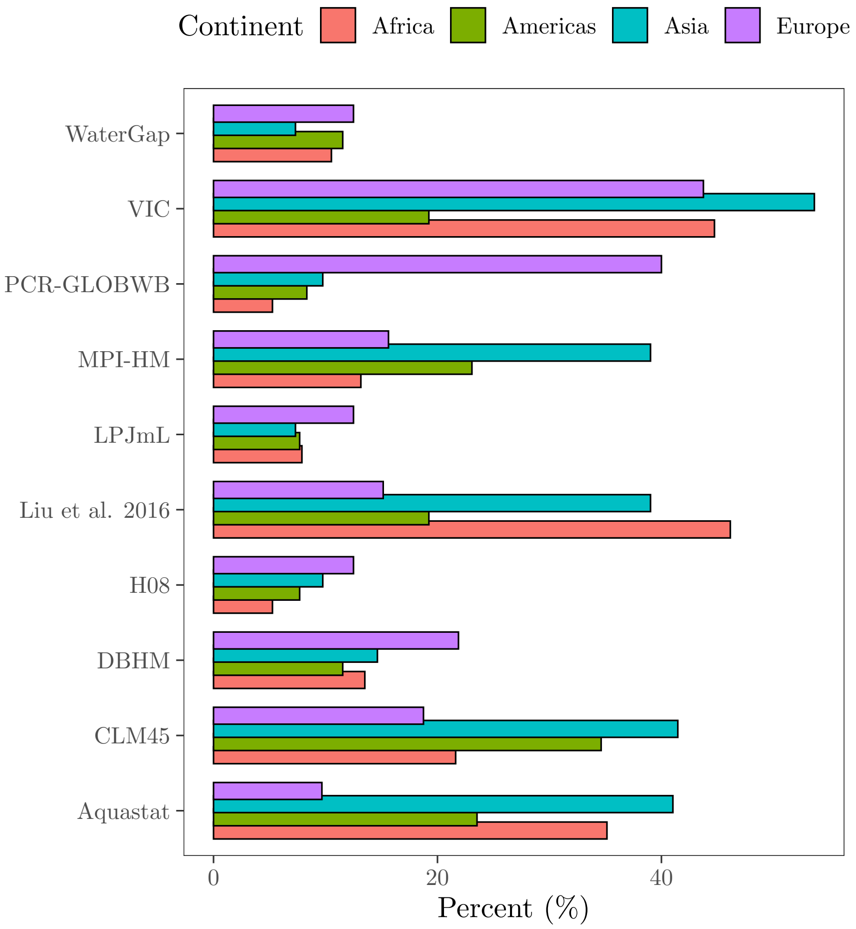

Figure S18: Percentage of countries with irrigation water withdrawal estimates beyond or above our range of predictions.

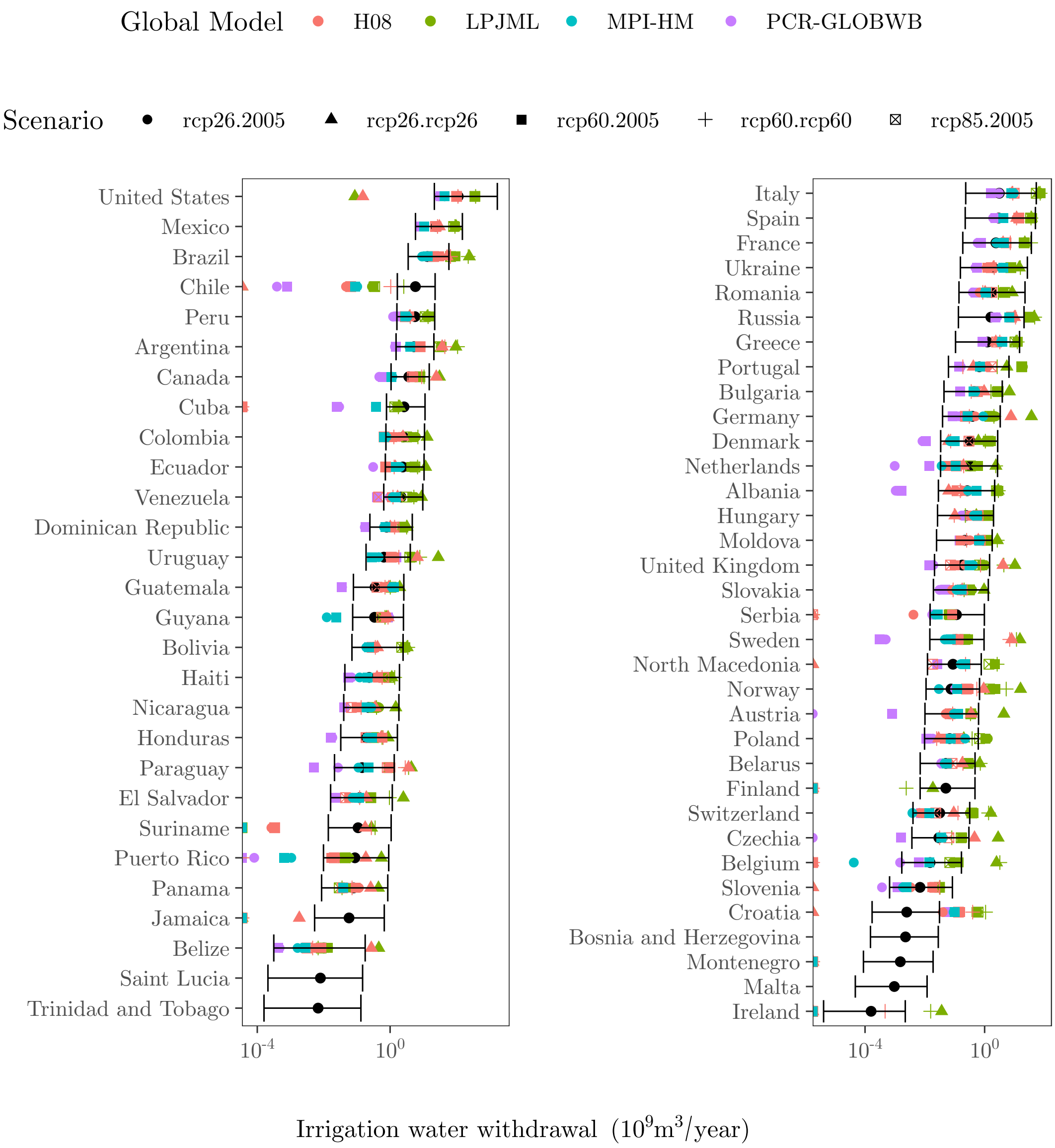

Figure S19: Comparison between our approach and the irrigation water withdrawals simulated by GM for 2050. The error bars and the black dots show the range and the median irrigation water withdrawal values obtained with our approach. The colored, shaped dots show the estimates produced by GM under five different social and climatic scenarios. Simulations with the "2005" suffix run on land use patterns (including the extension of irrigation) fixed at their 2005 values. Simulations that do not have the "2005" suffix assume that irrigated areas change according to the Shared Socioeconomic Pathway 2 (SSP2, "Middle of the Road"). `rcp26`, `rcp60` and `rcp85` refer to the Representative Concentration Pathway 2.6, 6 and 8.5 respectively [5].

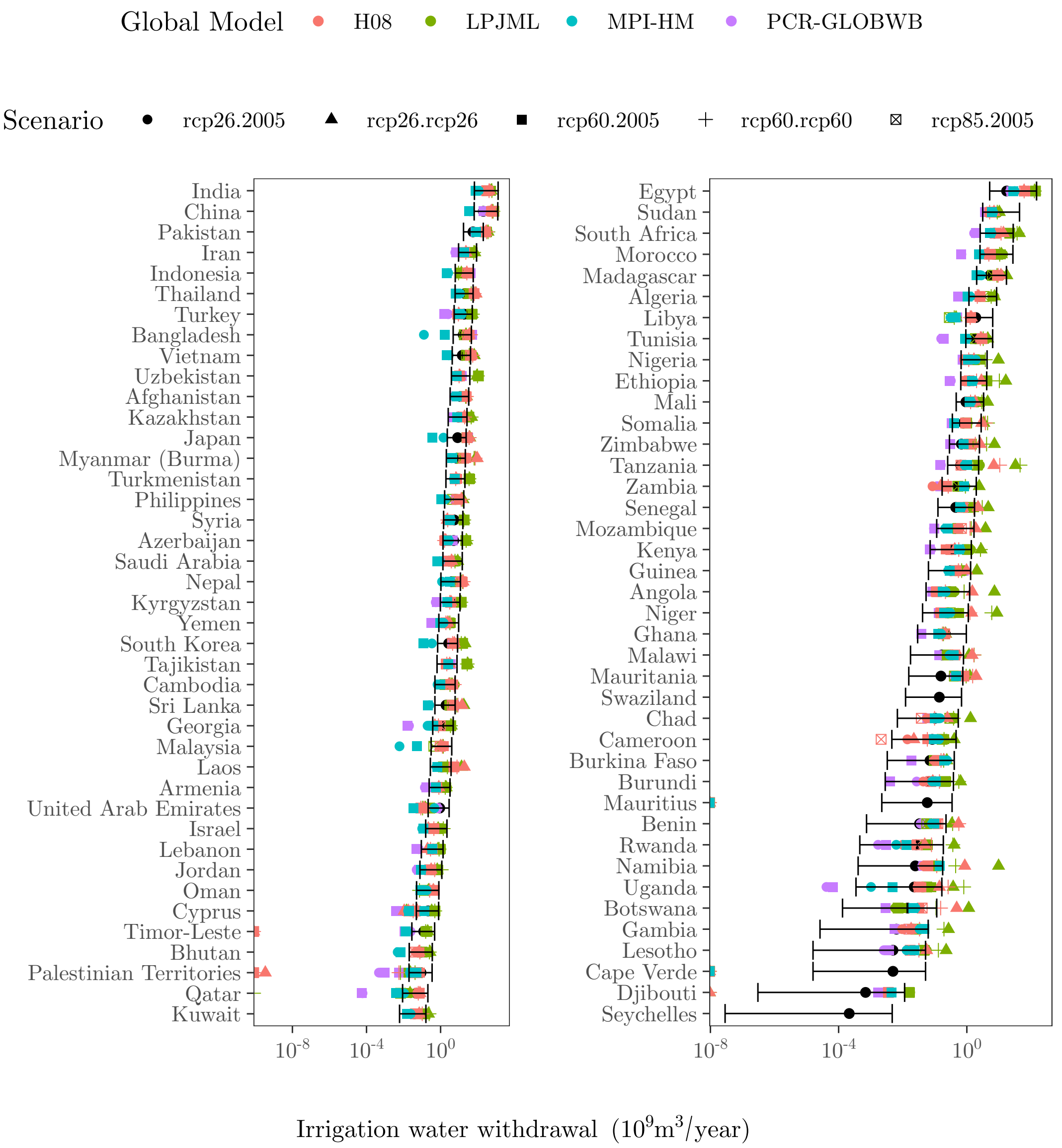

Figure S20: Comparison between our approach and the irrigation water withdrawals simulated by GM for 2050. The error bars and the black dots show the range and the median irrigation water withdrawal values obtained with our approach. The colored, shaped dots show the estimates produced by GM under five different social and climatic scenarios. Simulations with the "2005" suffix run on land use patterns (including the extension of irrigation) fixed at their 2005 values. Simulations that do not have the "2005" suffix assume that irrigated areas change according to the Shared Socioeconomic Pathway 2 (SSP2, "Middle of the Road"). `rcp26`, `rcp60` and `rcp85` refer to the Representative Concentration Pathway 2.6, 6 and 8.5 respectively [5].

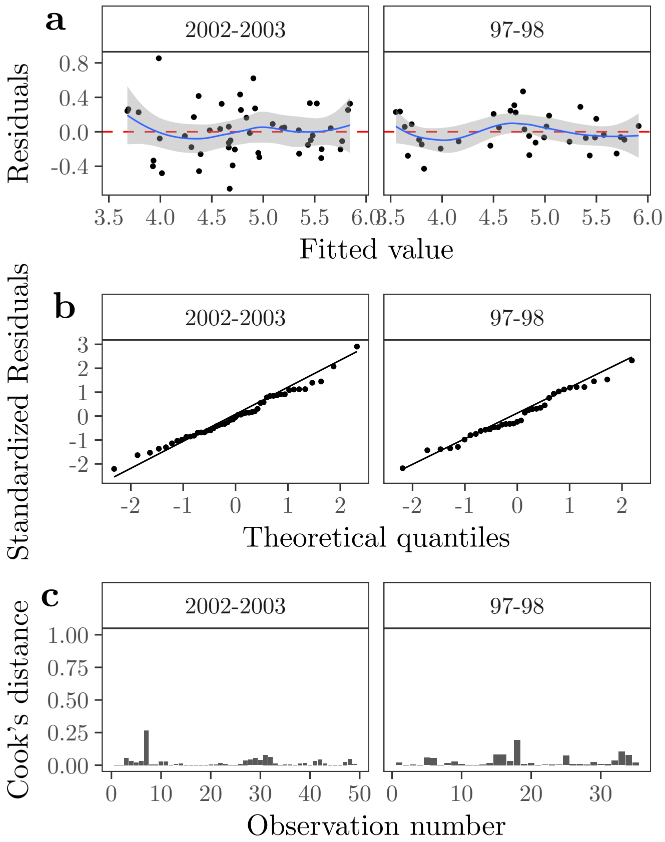

Figure S21: Regression diagnostics for the Australian irrigation systems regressions. a) Residuals versus fitted plot. b) Q-Q plot. c) Cook's distance plot.

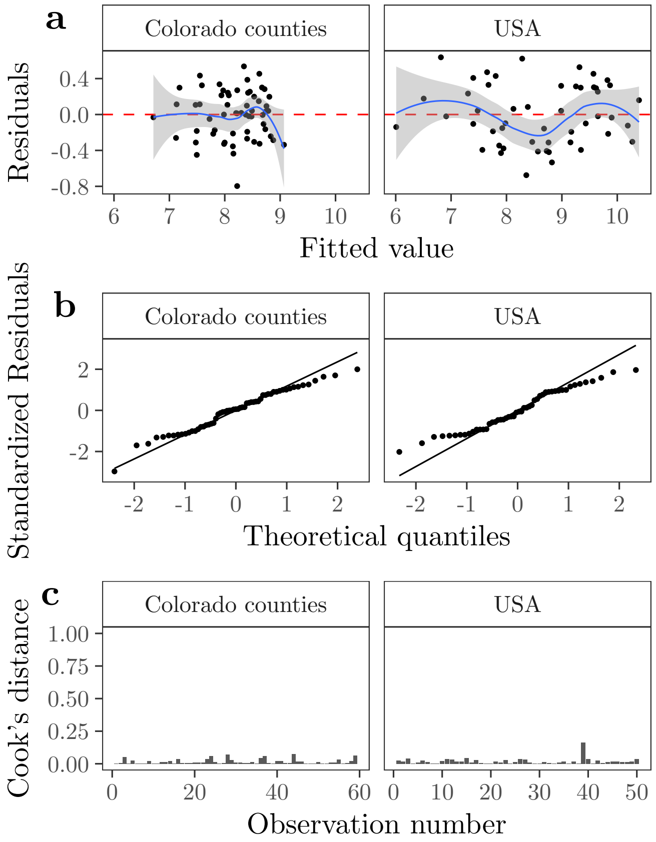

Figure S22: Diagnostics for the regressions conducted with the Colorado counties and the USA data. a) Residuals versus fitted plot. b) Q-Q plot. c) Cook's distance plot.

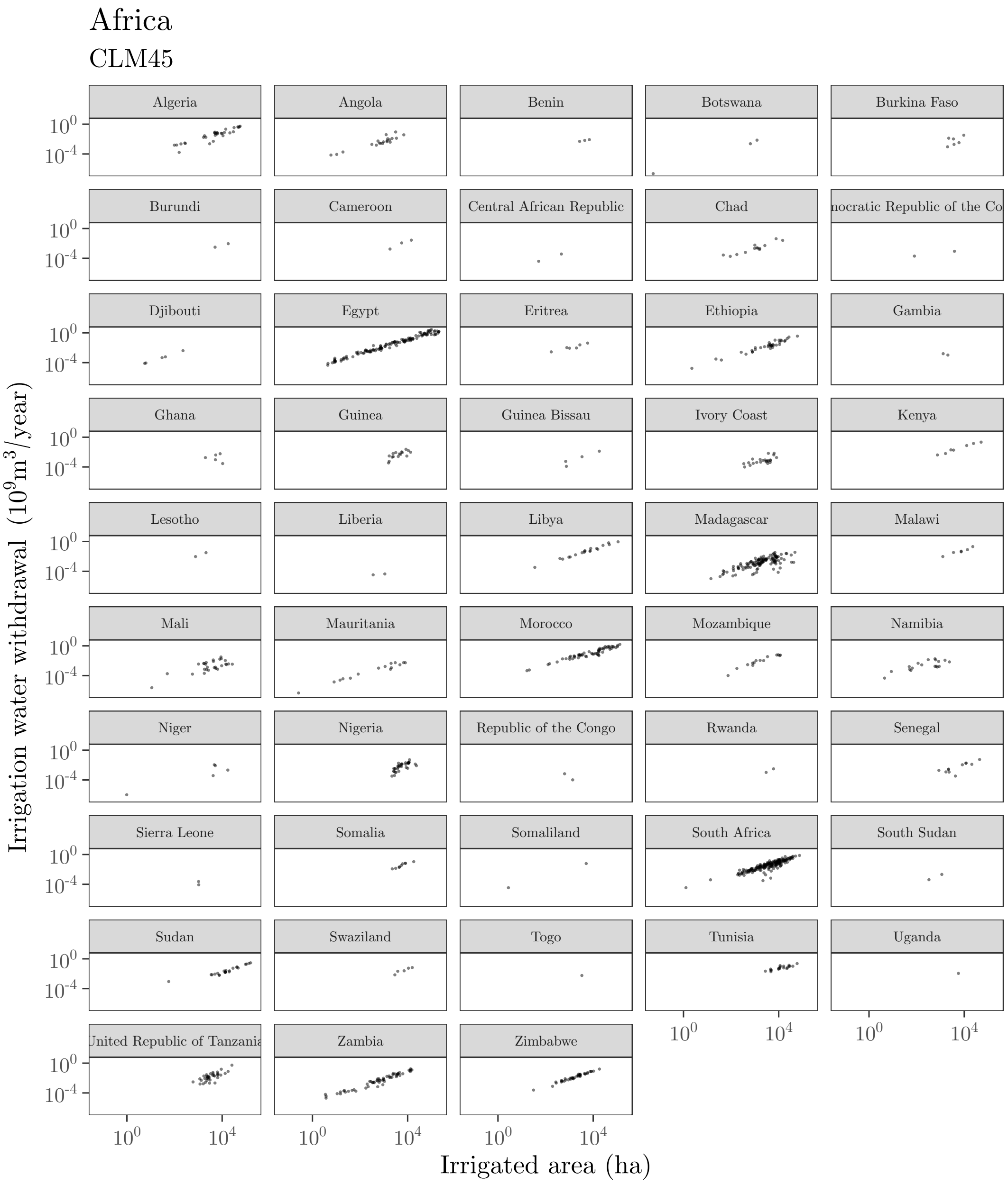

Figure S23: Irrigation water withdrawal against irrigated areas at the cell level. The data are retrieved from HYDE 3.2 [6]. Each dot is a cell.

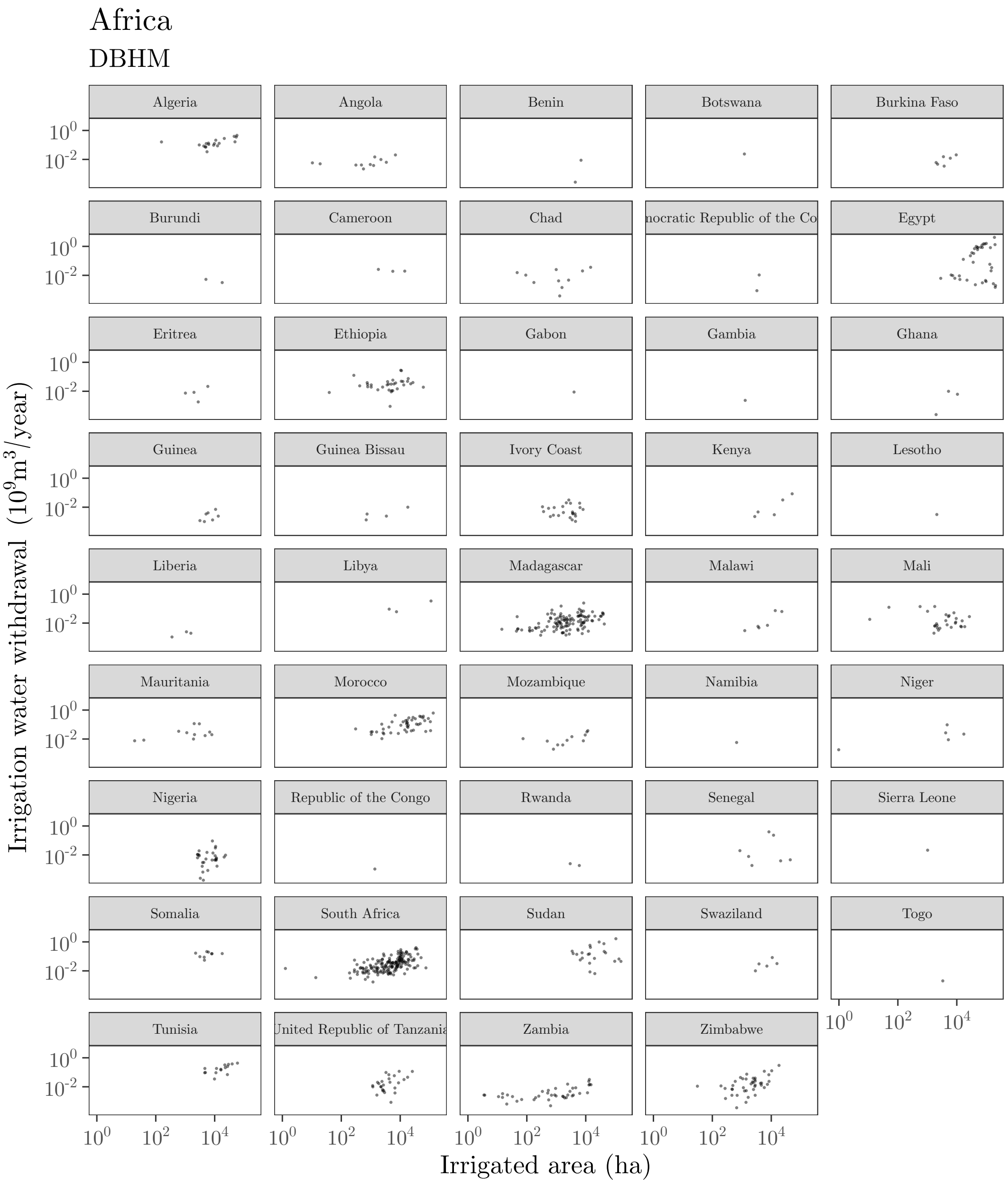

Figure S24: Irrigation water withdrawal against irrigated areas at the cell level. The data are retrieved from HYDE 3.2 [6]. Each dot is a cell.

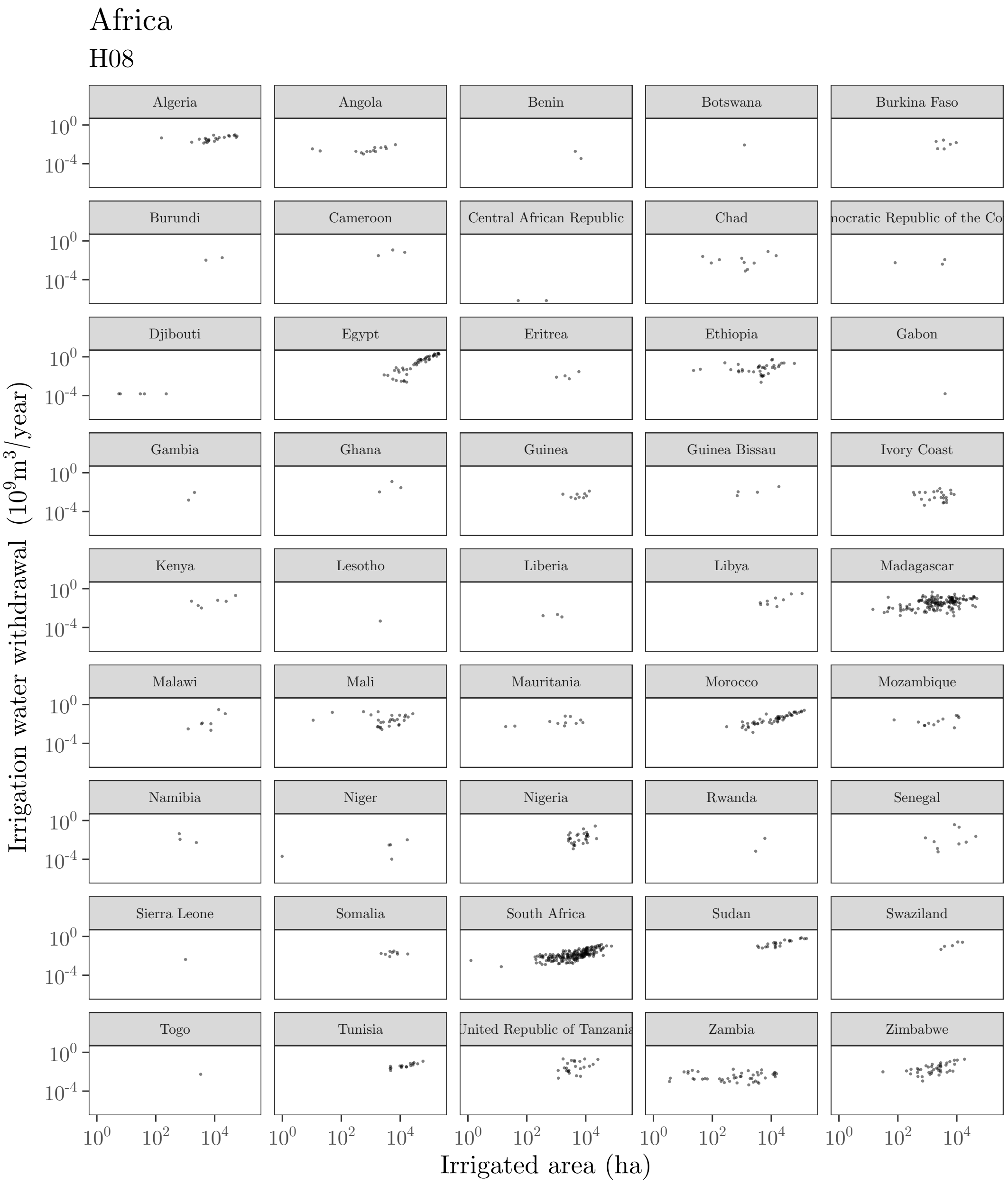

Figure S25: Irrigation water withdrawal against irrigated areas at the cell level. The data are retrieved from HYDE 3.2 [6]. Each dot is a cell.

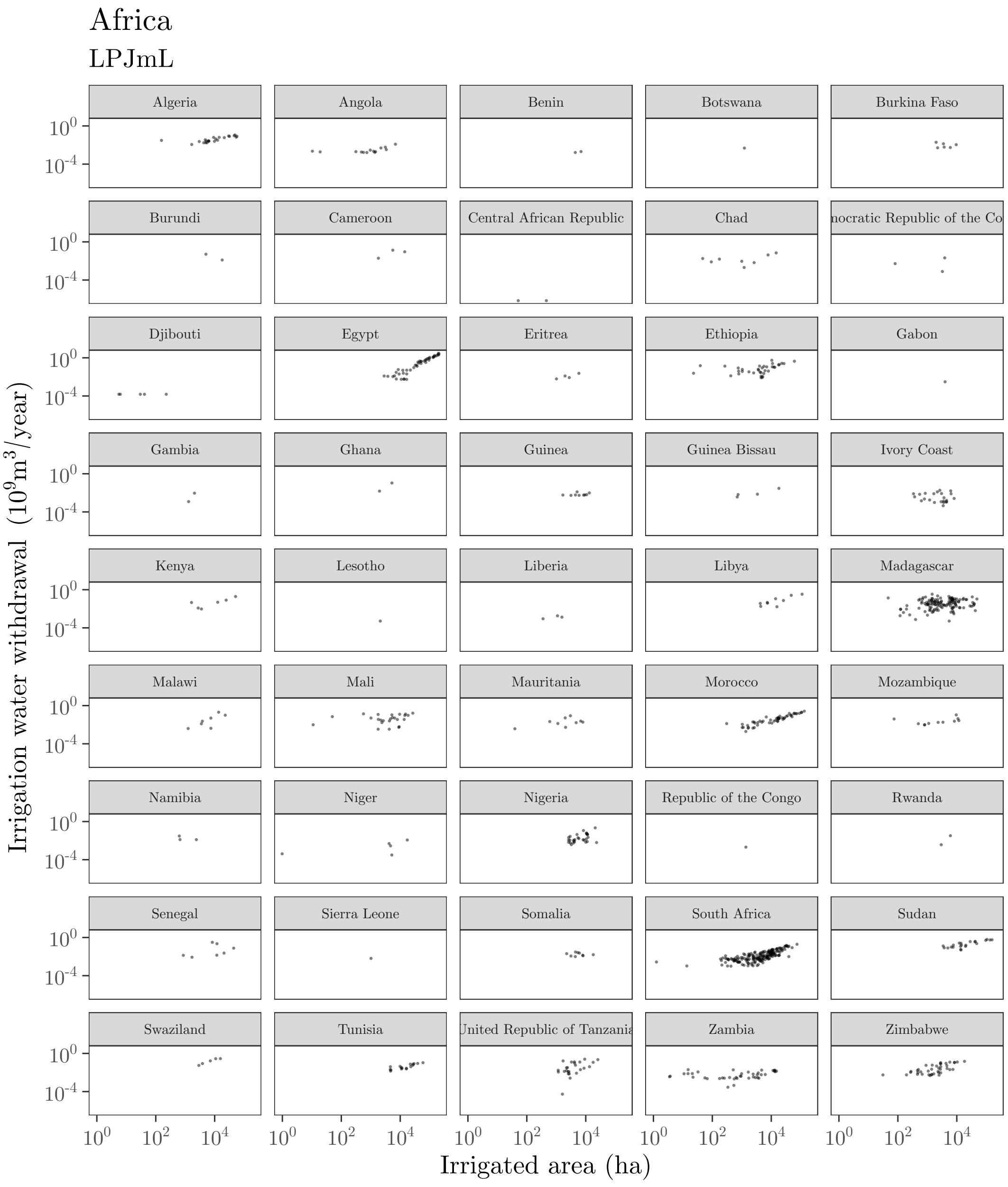

Figure S26: Irrigation water withdrawal against irrigated areas at the cell level. The data are retrieved from HYDE 3.2 [6]. Each dot is a cell.

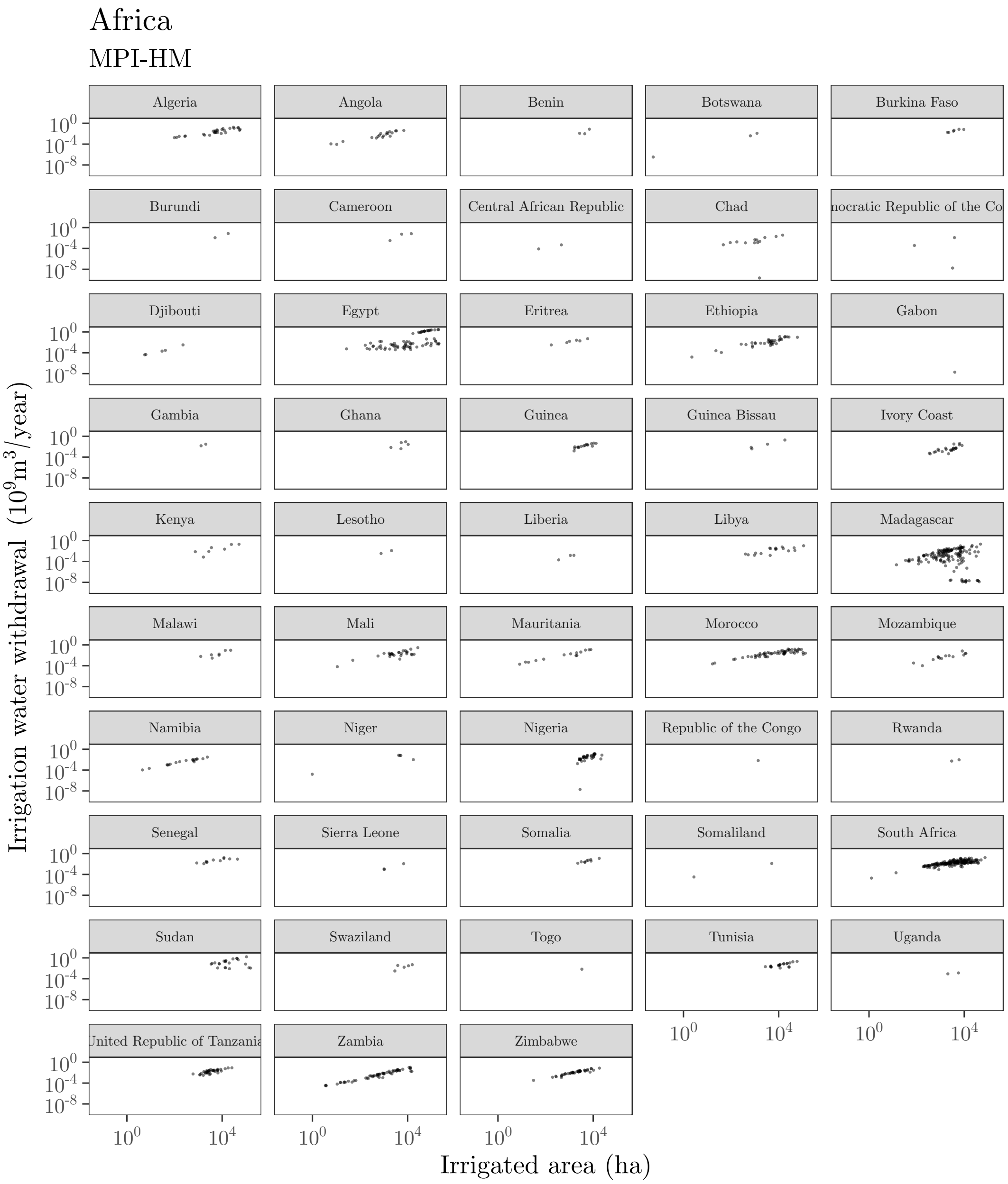

Figure S27: Irrigation water withdrawal against irrigated areas at the cell level. The data are retrieved from HYDE 3.2 [6]. Each dot is a cell.

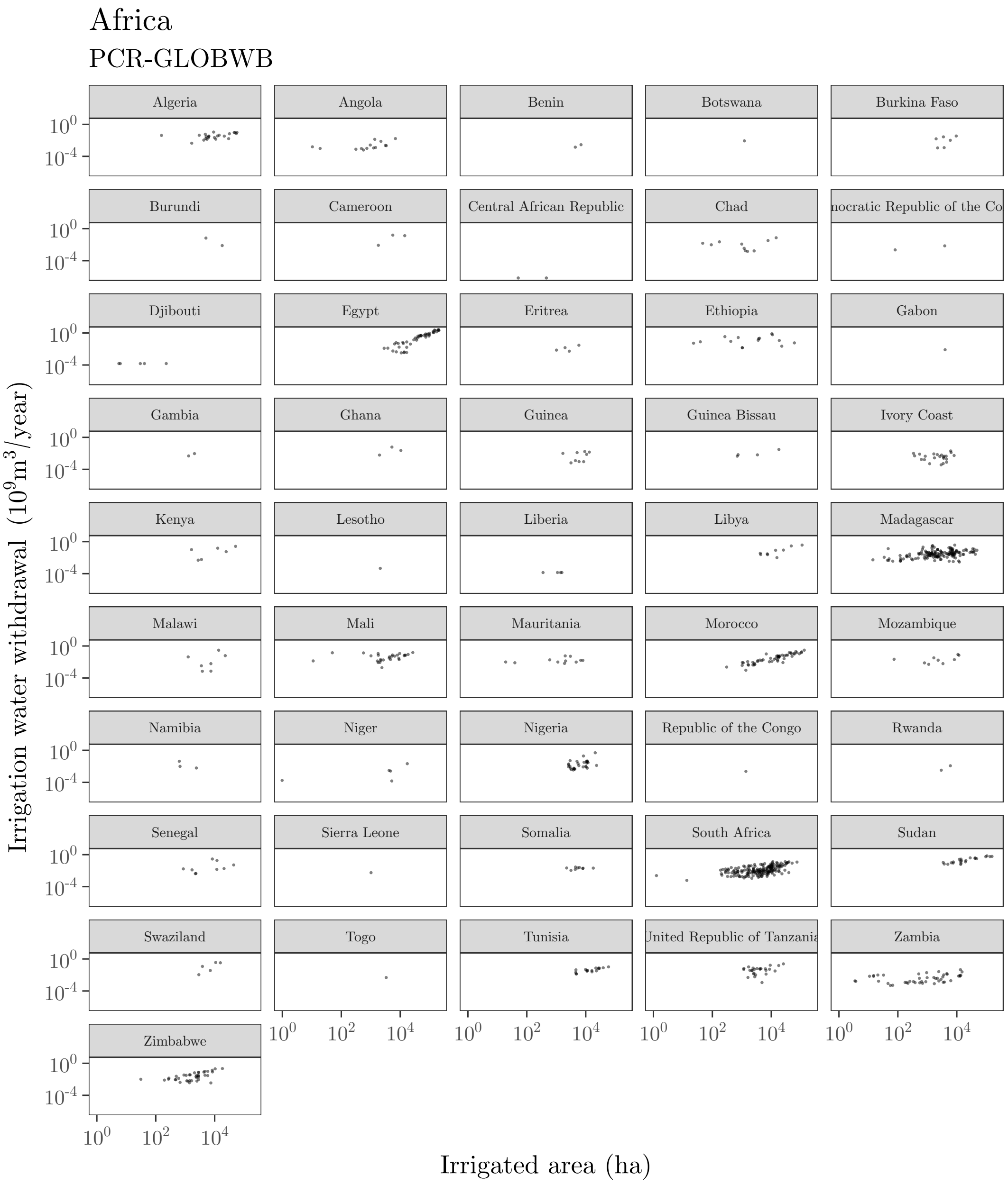

Figure S28: Irrigation water withdrawal against irrigated areas at the cell level. The data are retrieved from HYDE 3.2 [6]. Each dot is a cell.

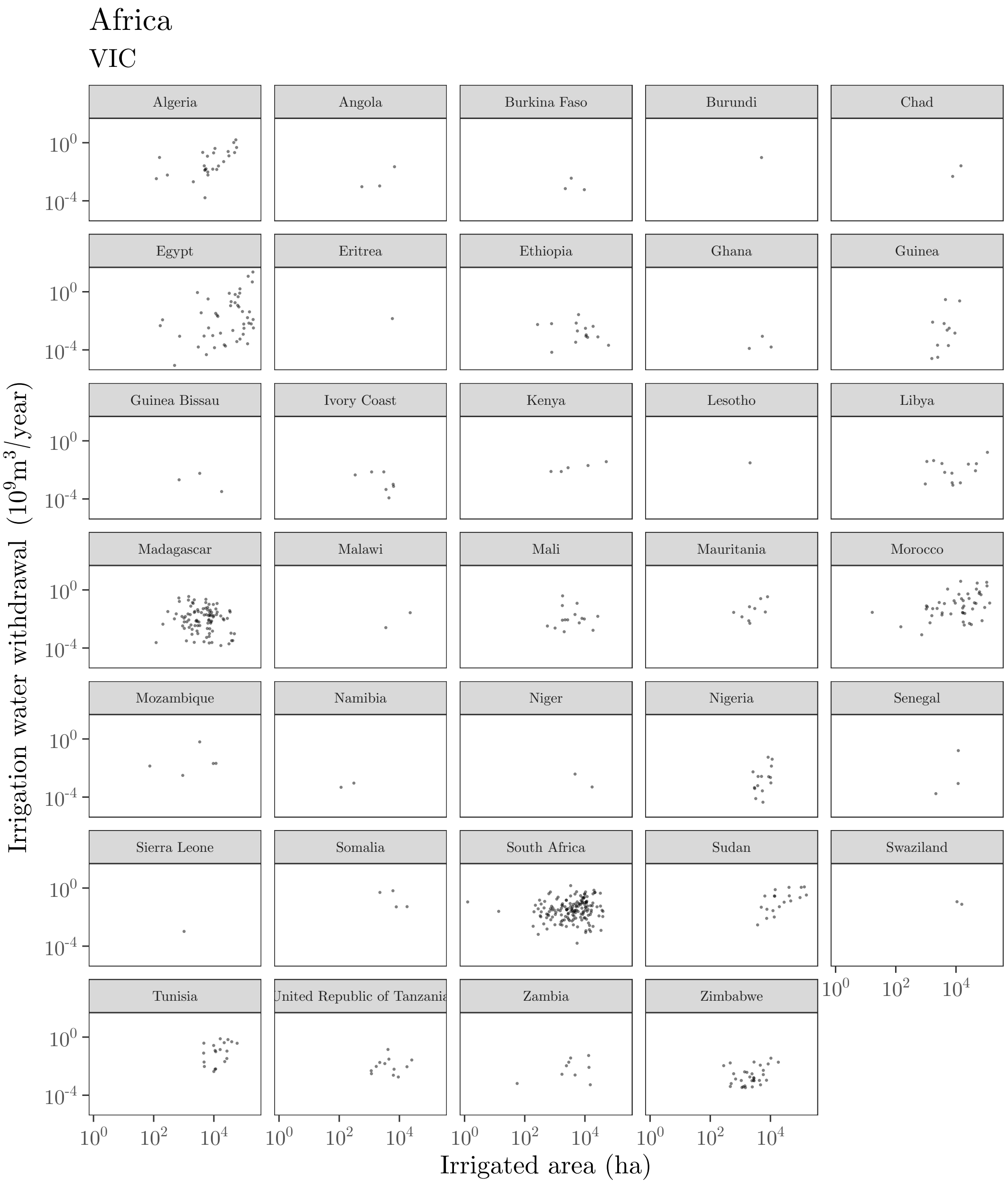

Figure S29: Irrigation water withdrawal against irrigated areas at the cell level. The data are retrieved from HYDE 3.2 [6]. Each dot is a cell.

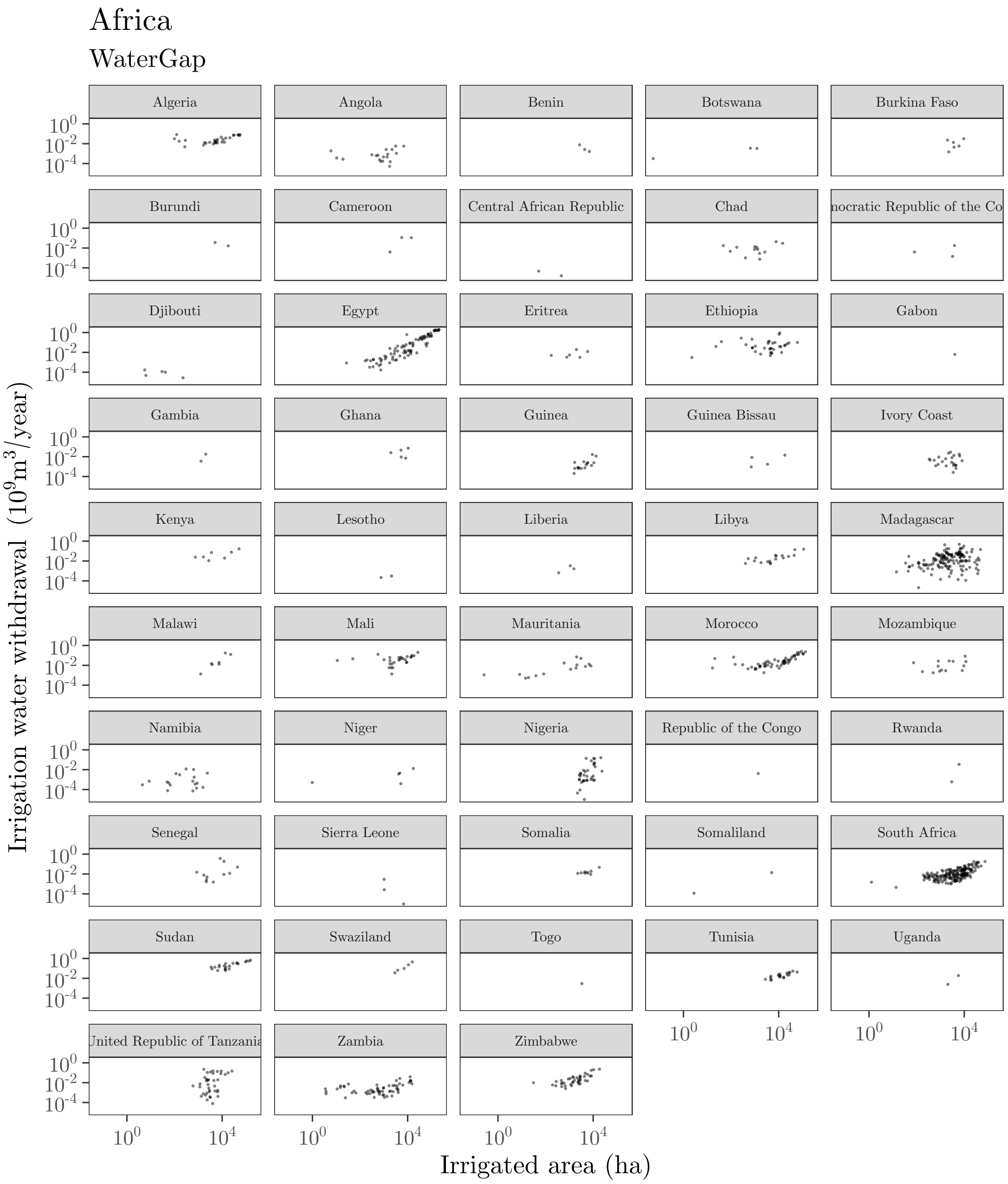

Figure S30: Irrigation water withdrawal against irrigated areas at the cell level. The data are retrieved from HYDE 3.2 [6]. Each dot is a cell.

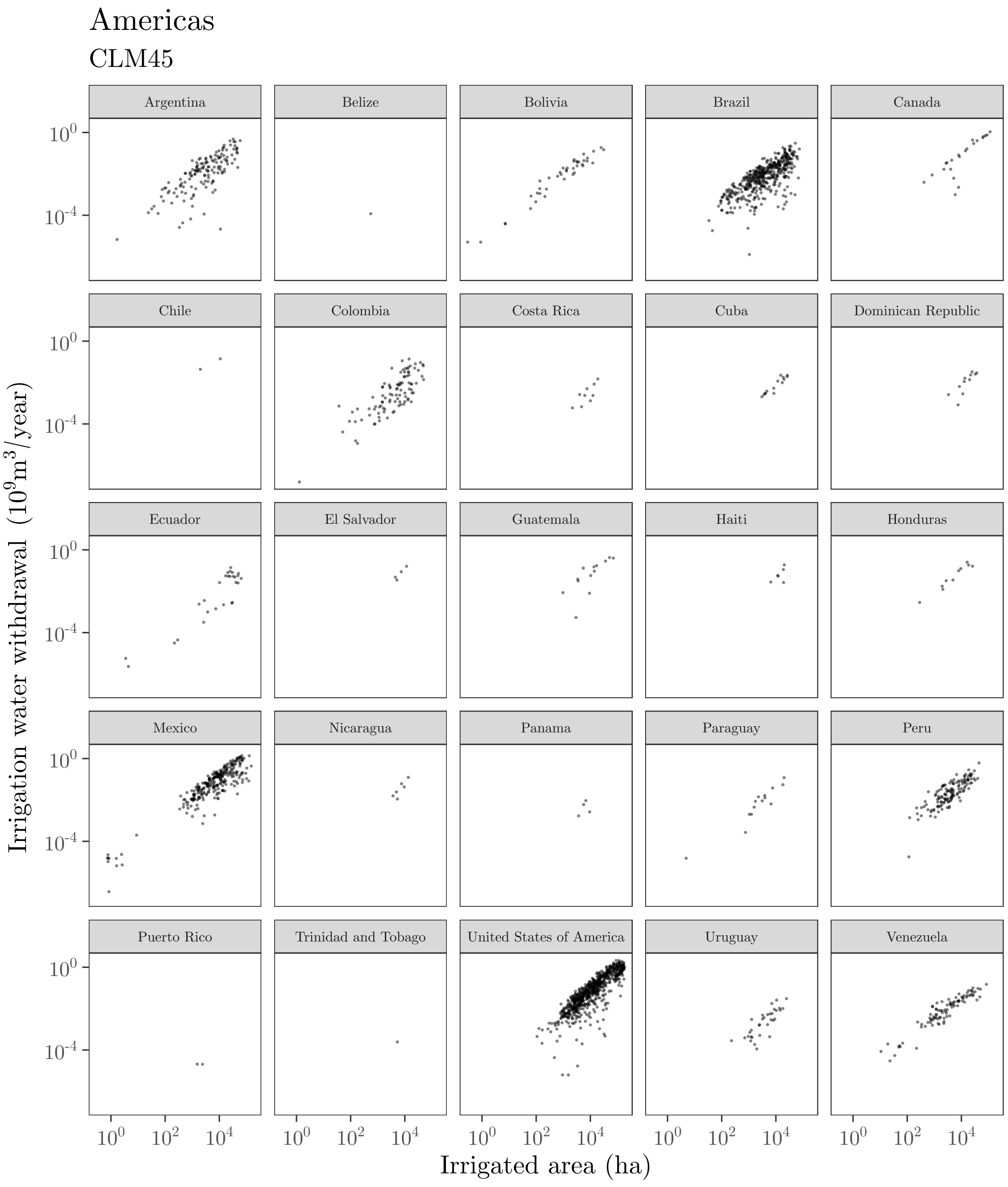

Figure S31: Irrigation water withdrawal against irrigated areas at the cell level. The data are retrieved from HYDE 3.2 [6]. Each dot is a cell.

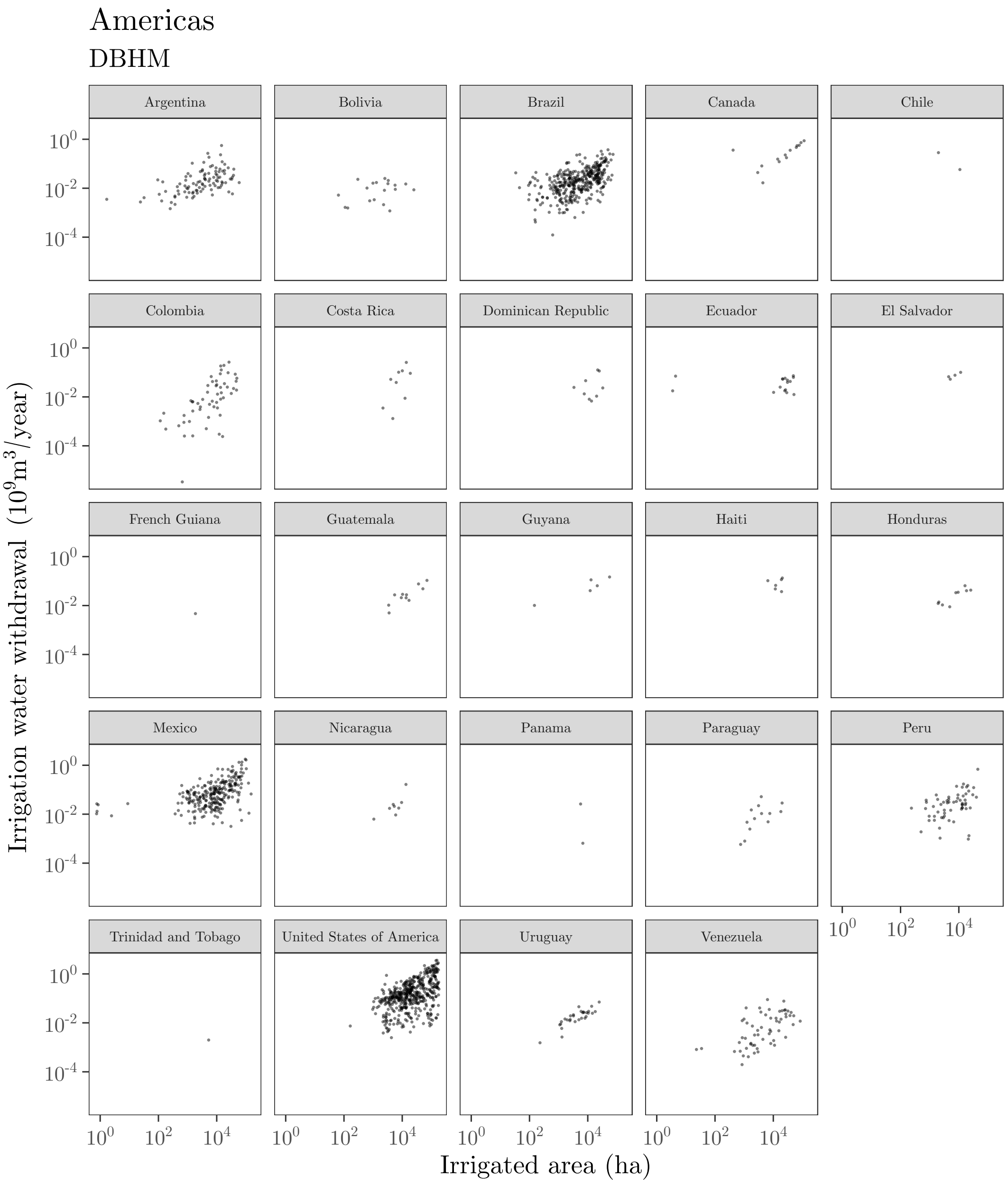

Figure S32: Irrigation water withdrawal against irrigated areas at the cell level. The data are retrieved from HYDE 3.2 [6]. Each dot is a cell.

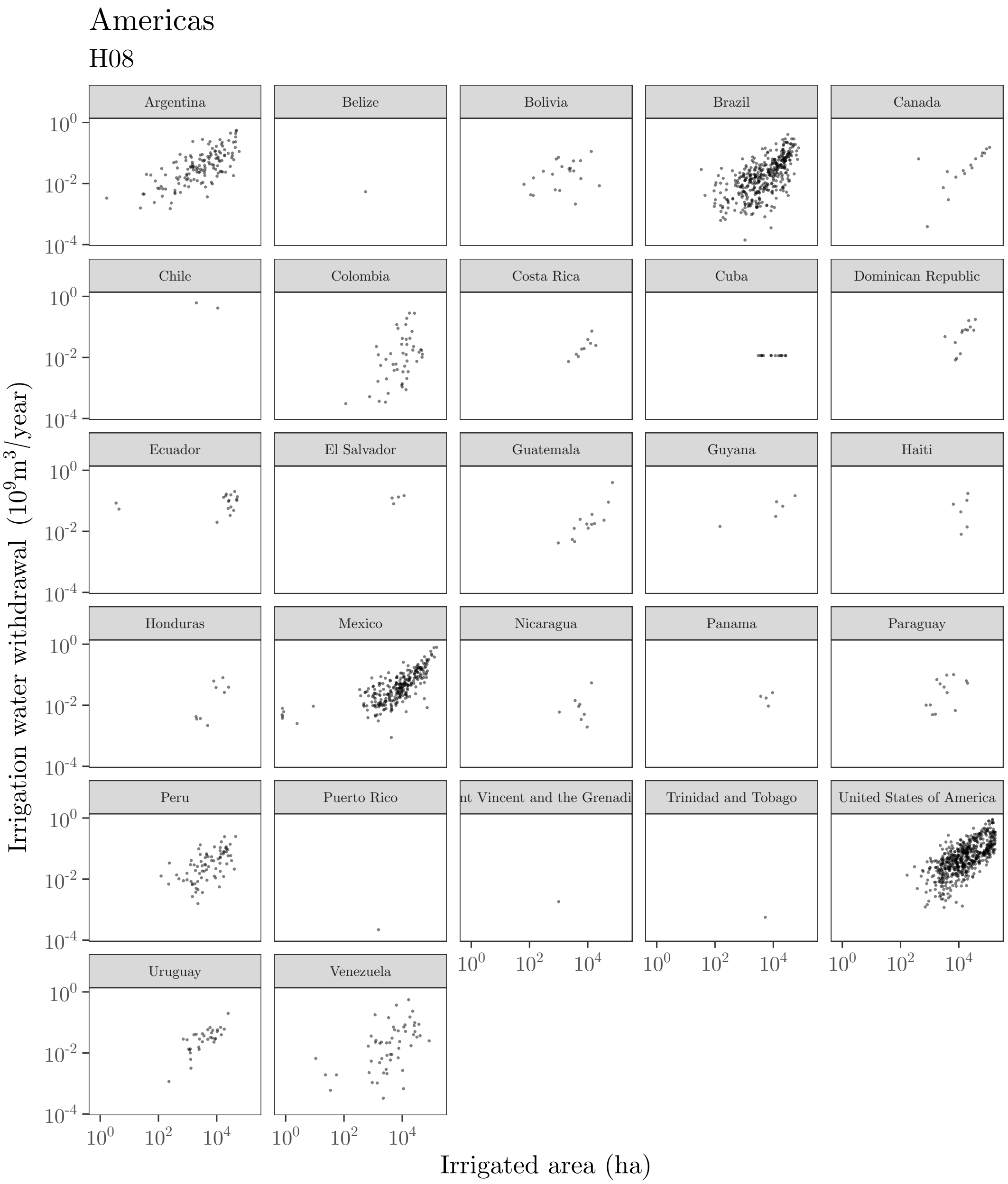

Figure S33: Irrigation water withdrawal against irrigated areas at the cell level. The data are retrieved from HYDE 3.2 [6]. Each dot is a cell.

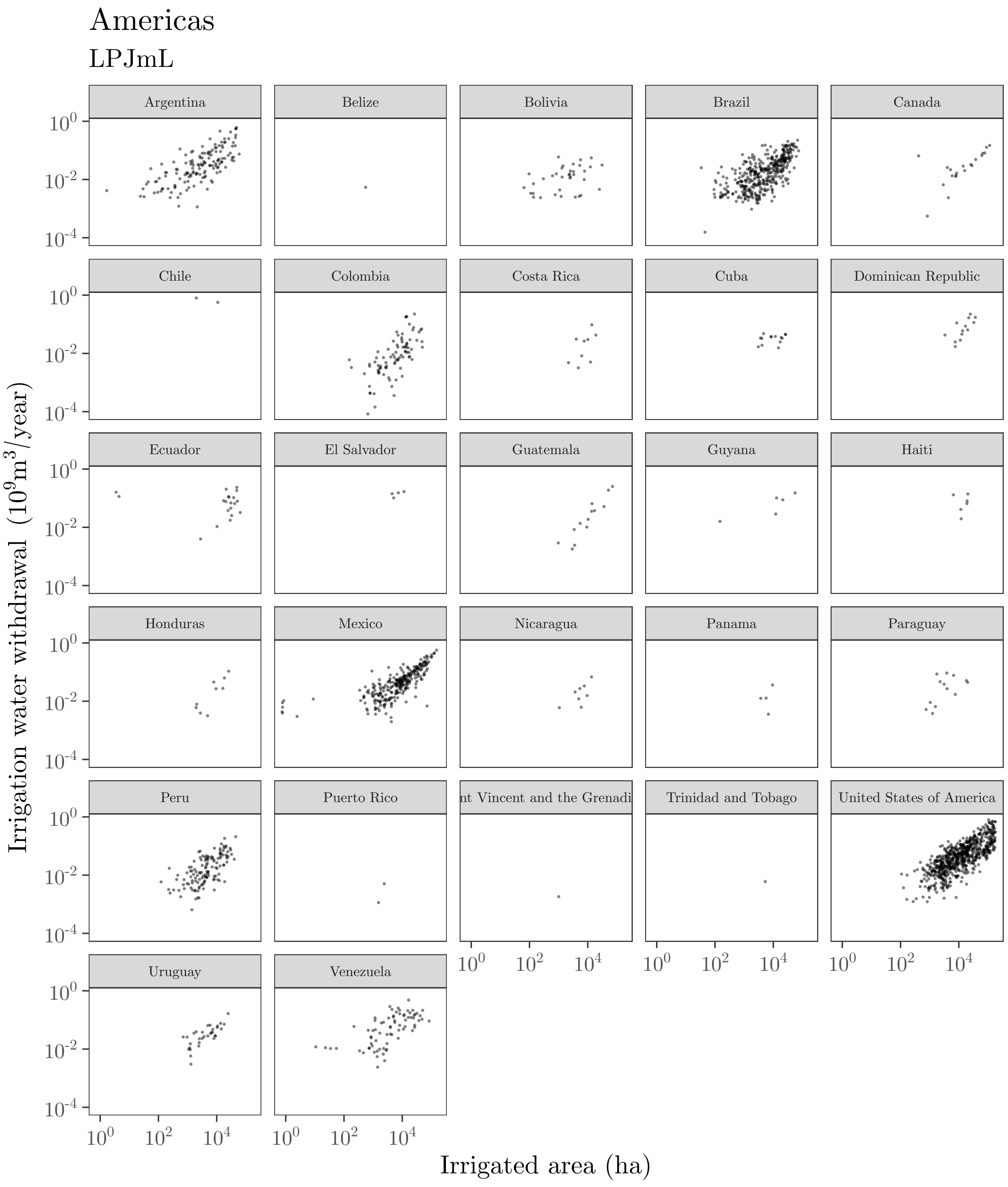

Figure S34: Irrigation water withdrawal against irrigated areas at the cell level. The data are retrieved from HYDE 3.2 [6]. Each dot is a cell.

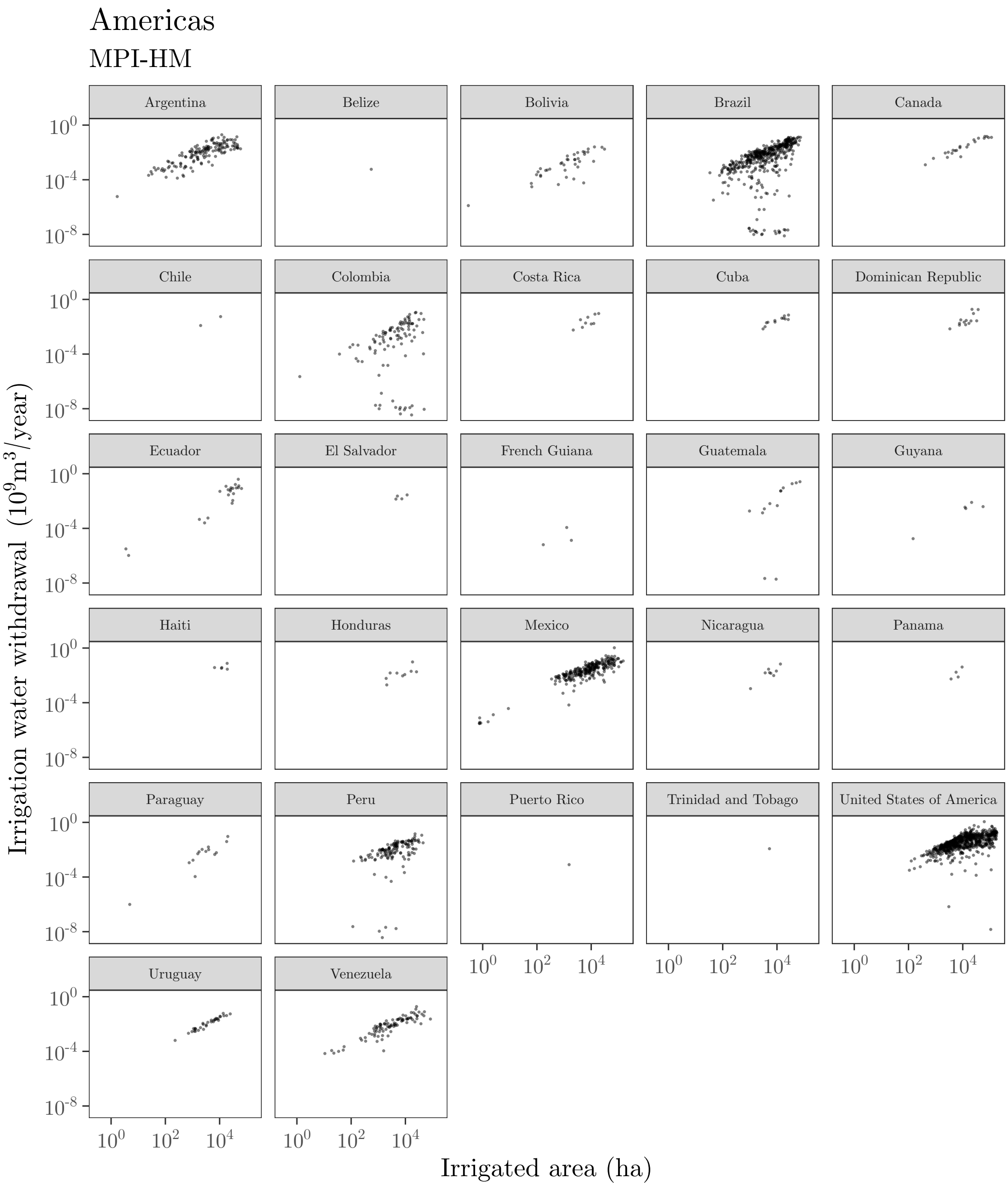

Figure S35: Irrigation water withdrawal against irrigated areas at the cell level. The data are retrieved from HYDE 3.2 [6]. Each dot is a cell.

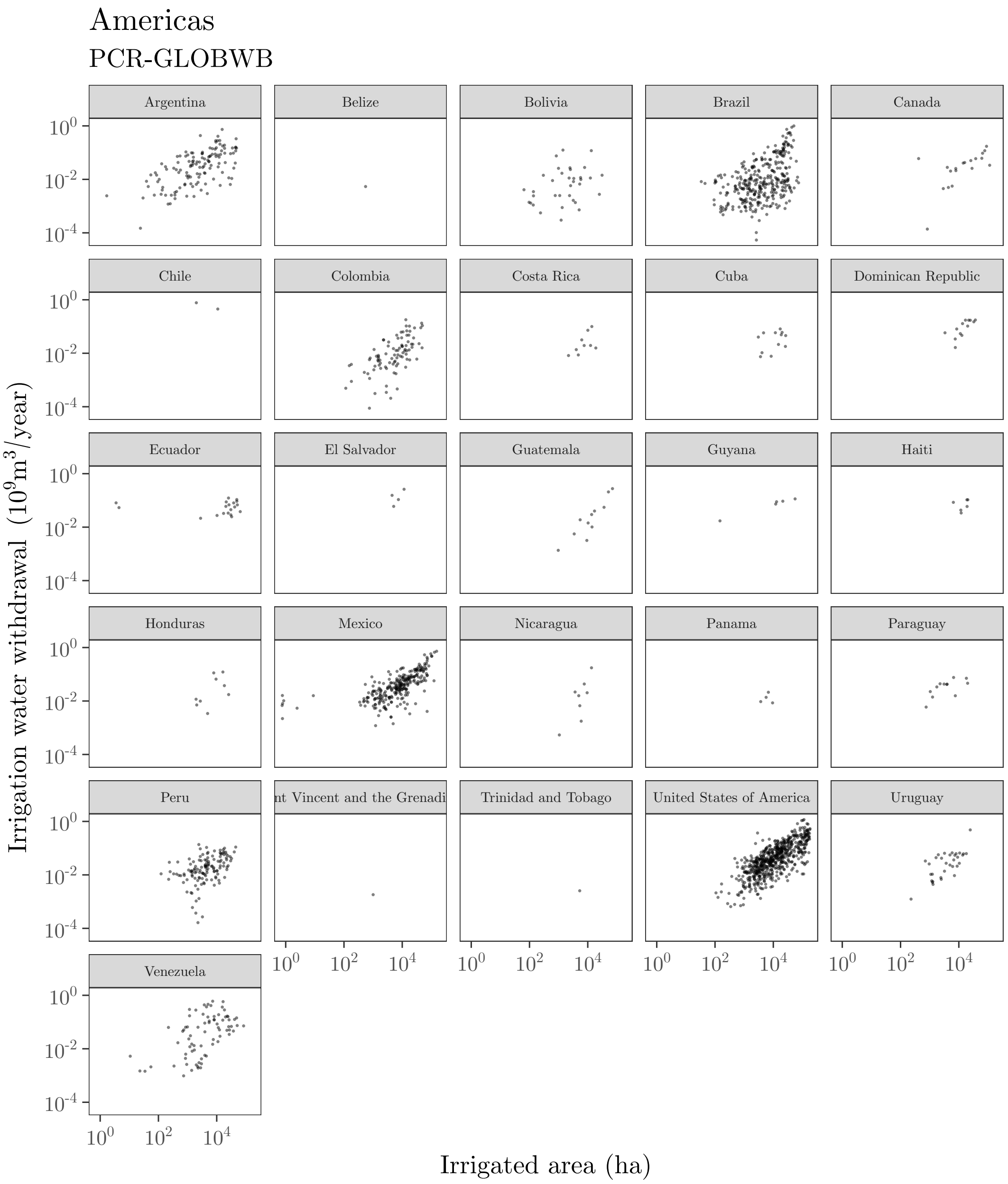

Figure S36: Irrigation water withdrawal against irrigated areas at the cell level. The data are retrieved from HYDE 3.2 [6]. Each dot is a cell.

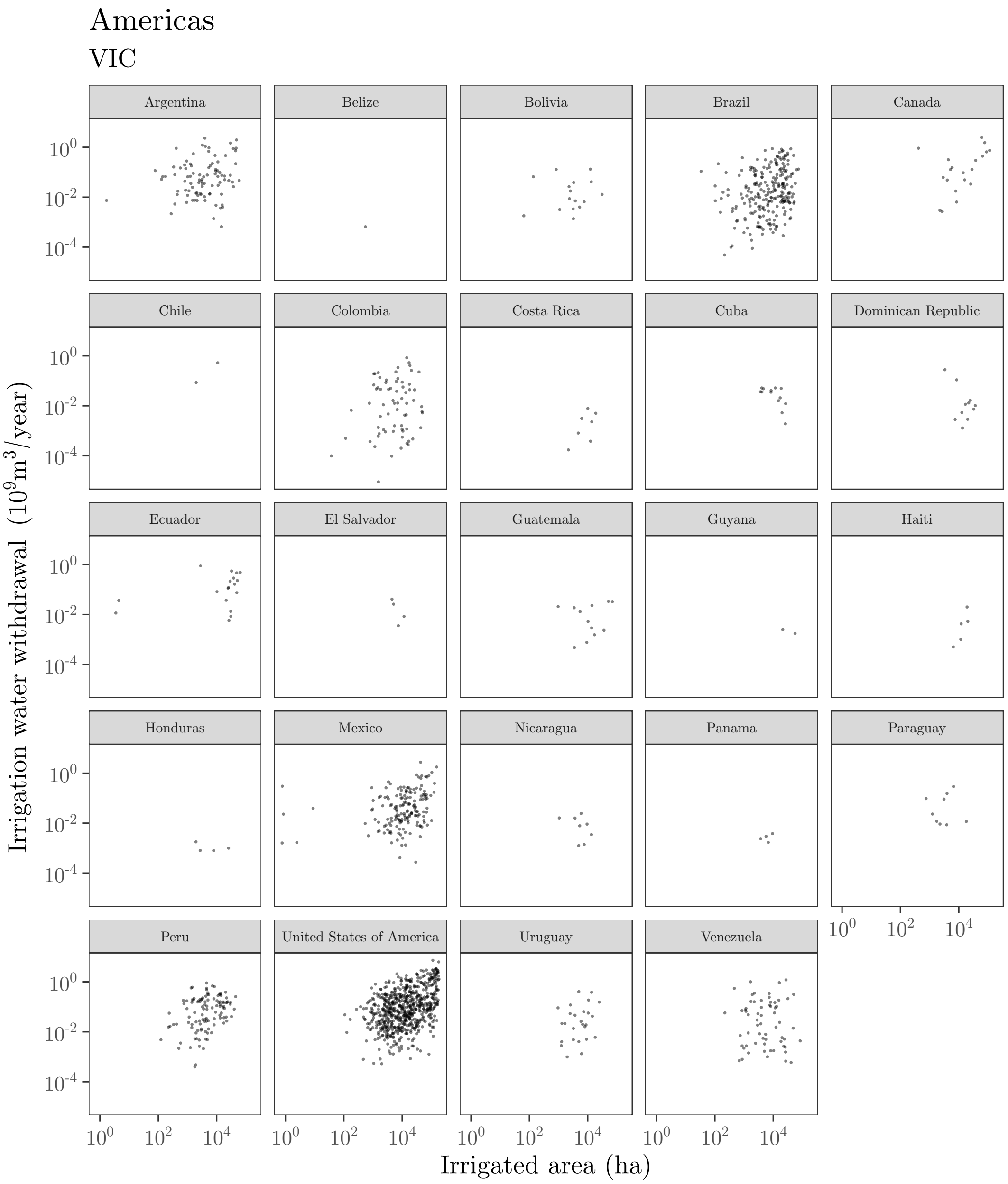

Figure S37: Irrigation water withdrawal against irrigated areas at the cell level. The data are retrieved from HYDE 3.2 [6]. Each dot is a cell.

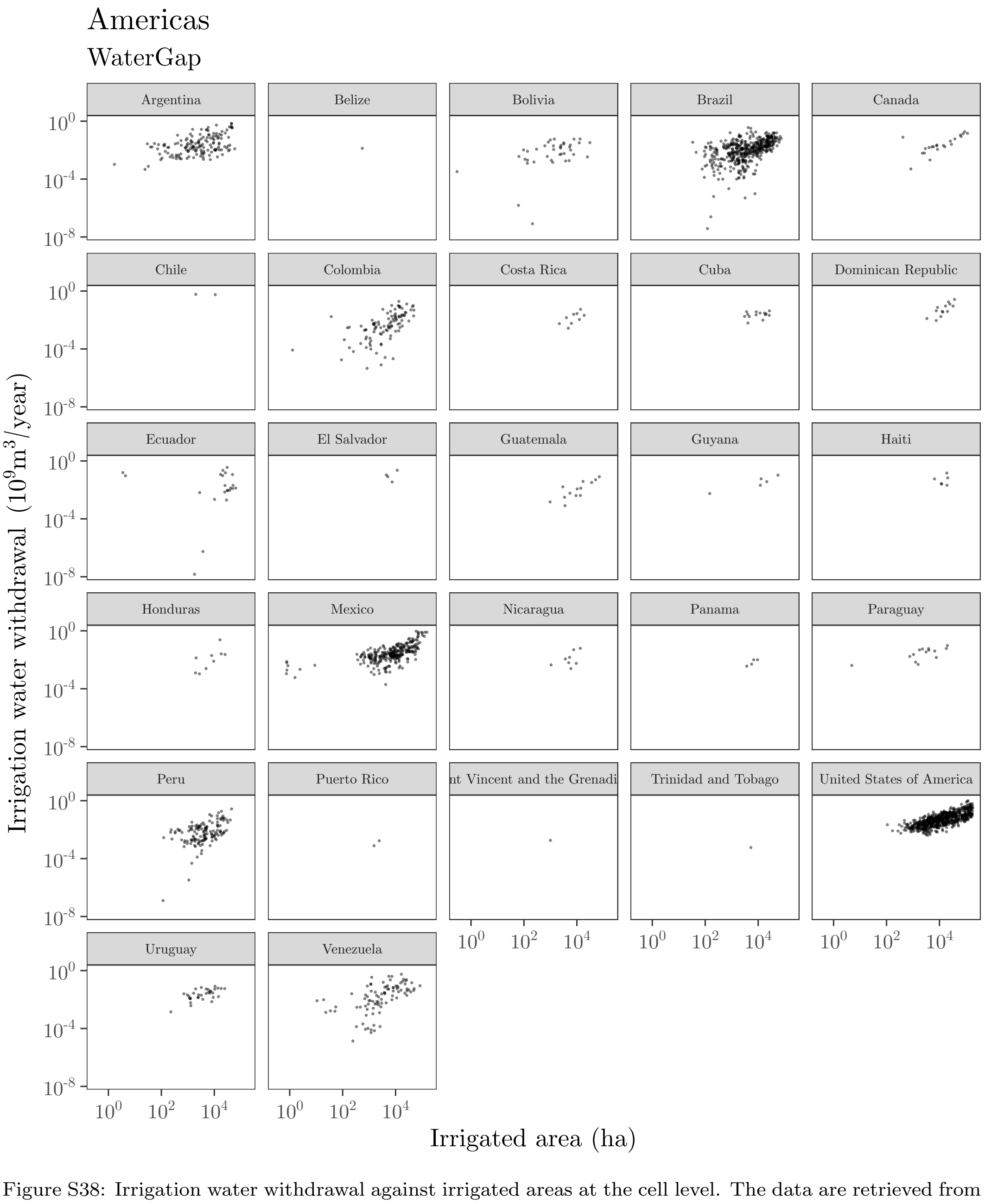

Figure S38: Irrigation water withdrawal against irrigated areas at the cell level. The data are retrieved from HYDE 3.2 [6]. Each dot is a cell.

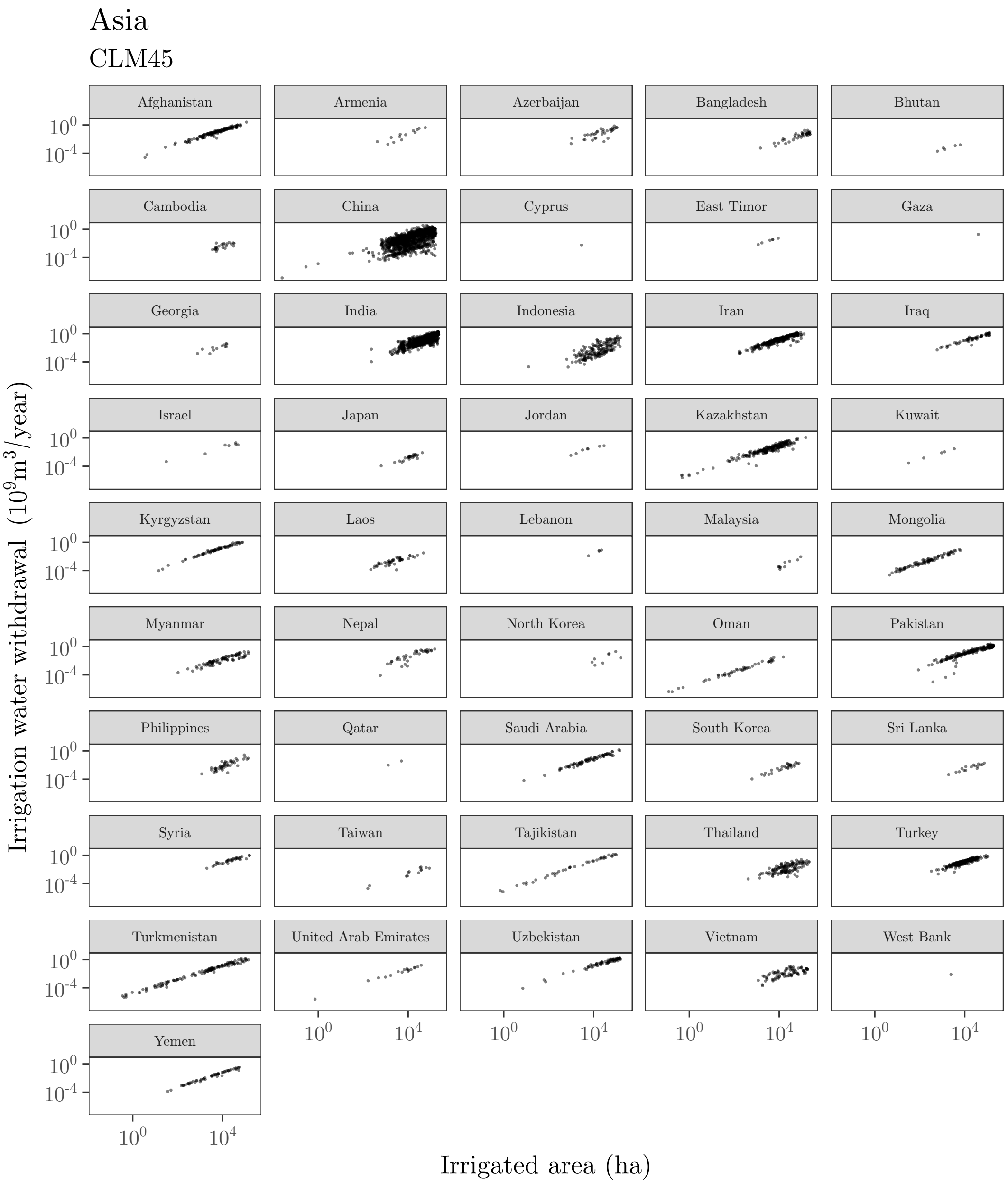

Figure S39: Irrigation water withdrawal against irrigated areas at the cell level. The data are retrieved from HYDE 3.2 [6]. Each dot is a cell.

Asia

DBHM

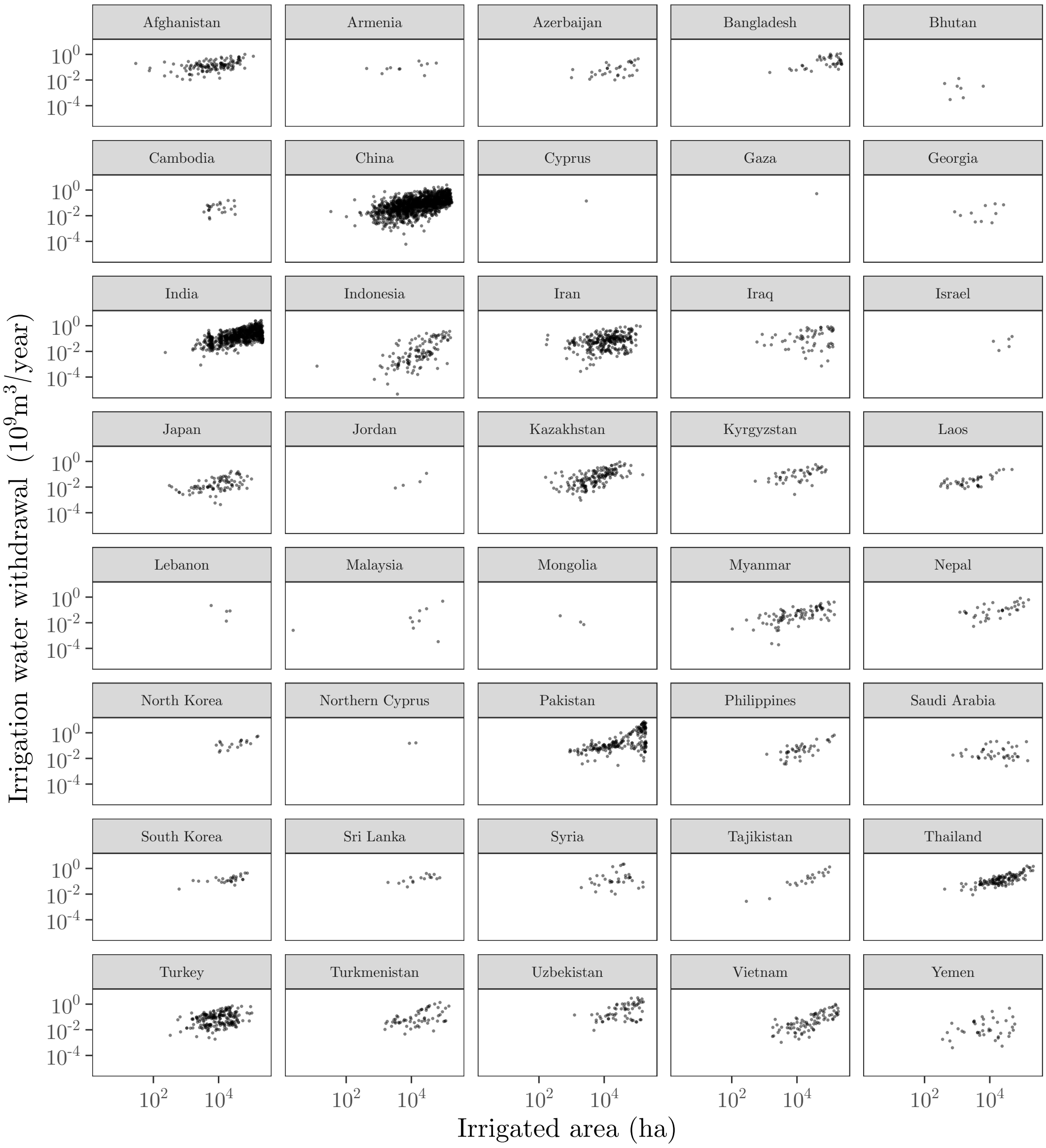

Figure S40: Irrigation water withdrawal against irrigated areas at the cell level. The data are retrieved from HYDE 3.2 [6]. Each dot is a cell.

Asia

H08

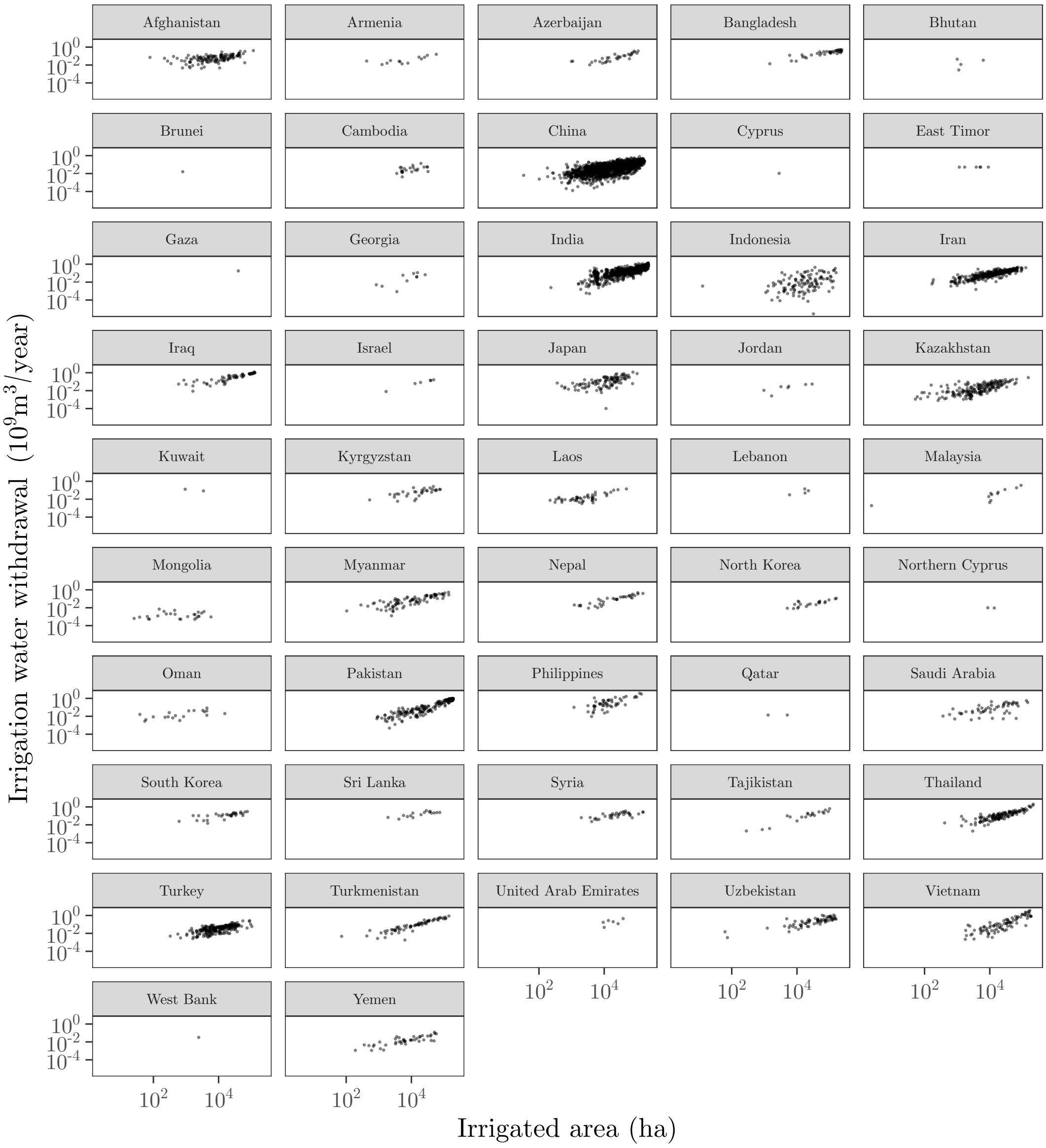

Figure S41: Irrigation water withdrawal against irrigated areas at the cell level. The data are retrieved from HYDE 3.2 [6]. Each dot is a cell.

Asia

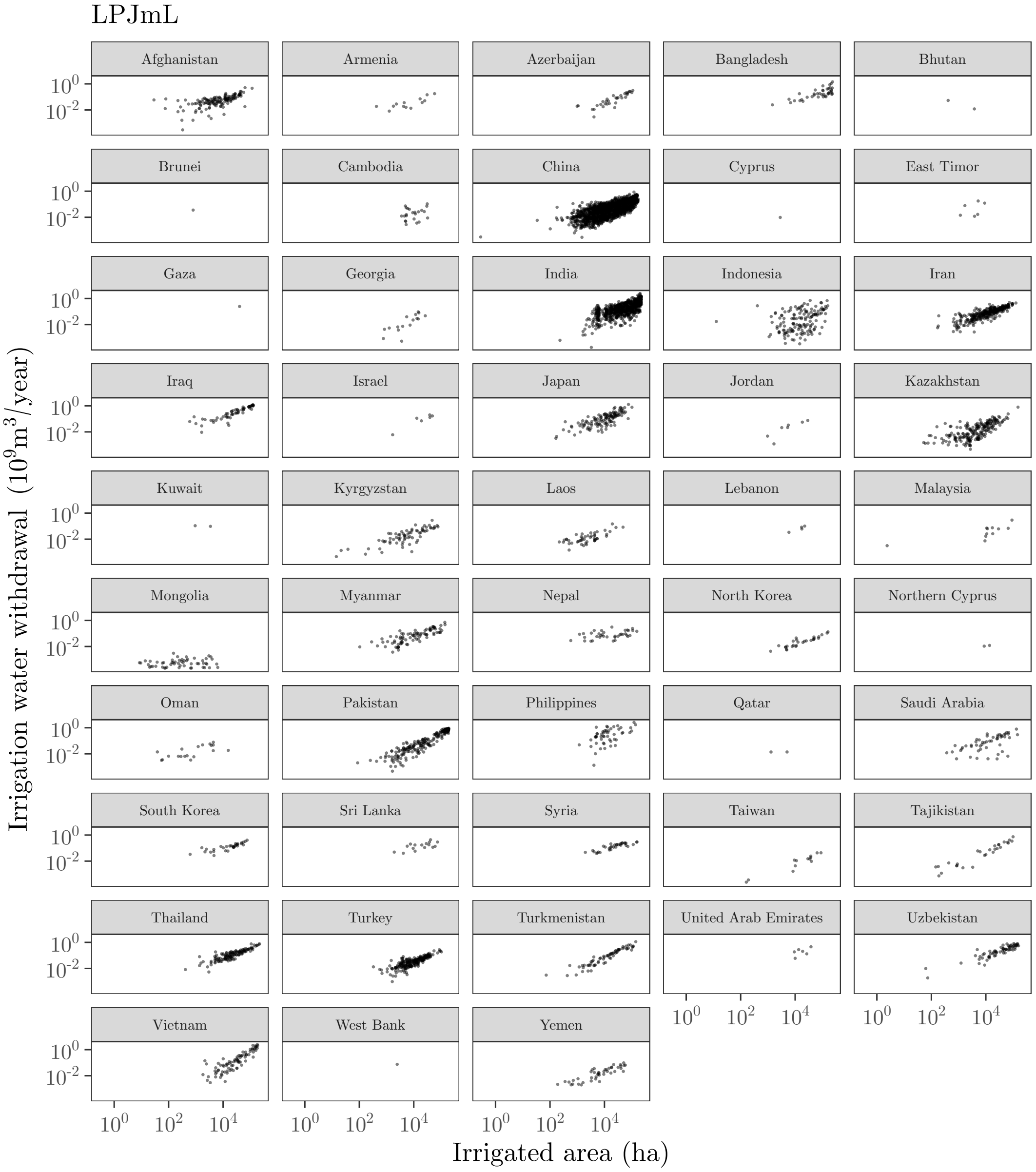

Figure S42: Irrigation water withdrawal against irrigated areas at the cell level. The data are retrieved from HYDE 3.2 [6]. Each dot is a cell.

Asia

MPI-HM

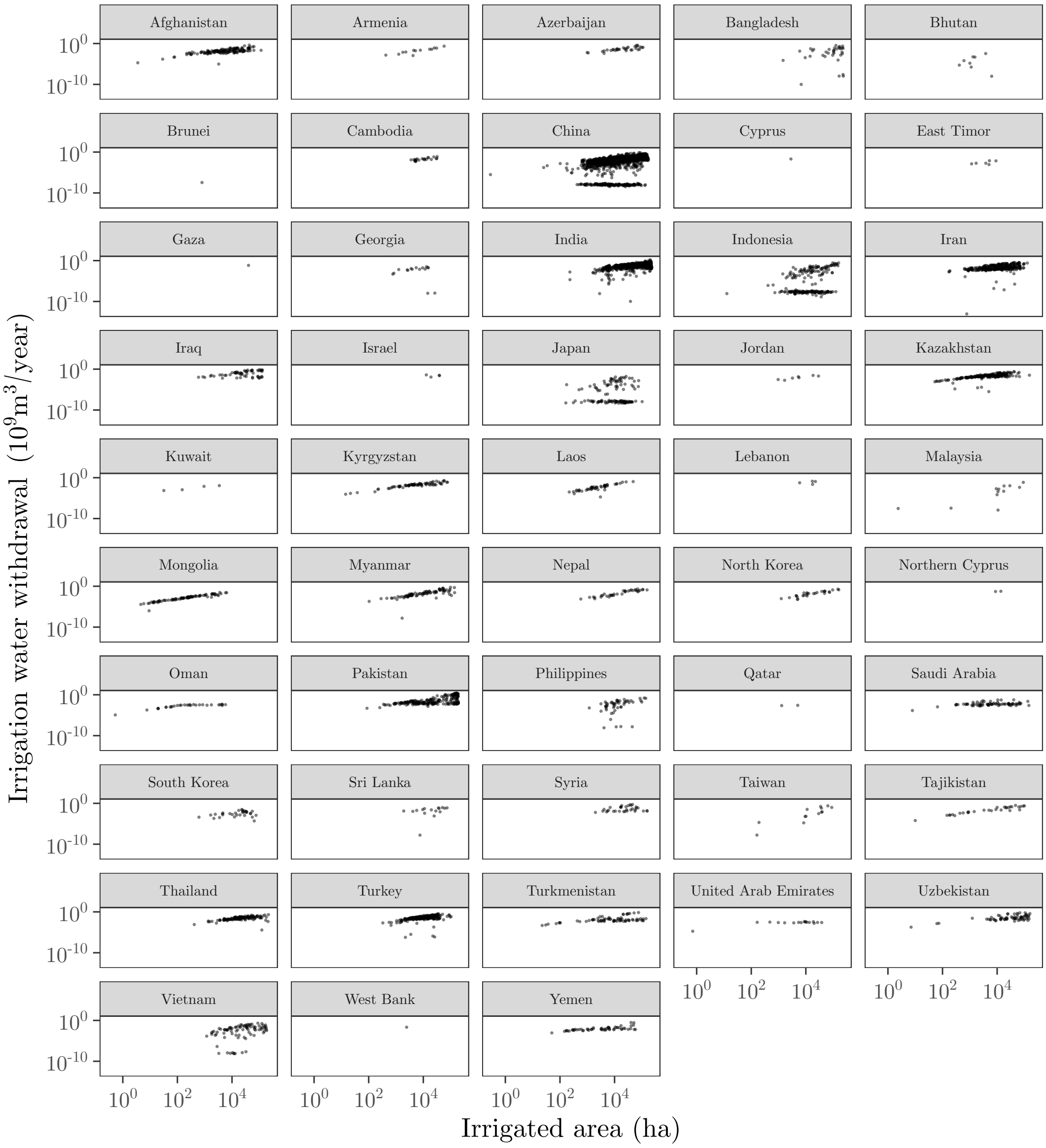

Figure S43: Irrigation water withdrawal against irrigated areas at the cell level. The data are retrieved from HYDE 3.2 [6]. Each dot is a cell.

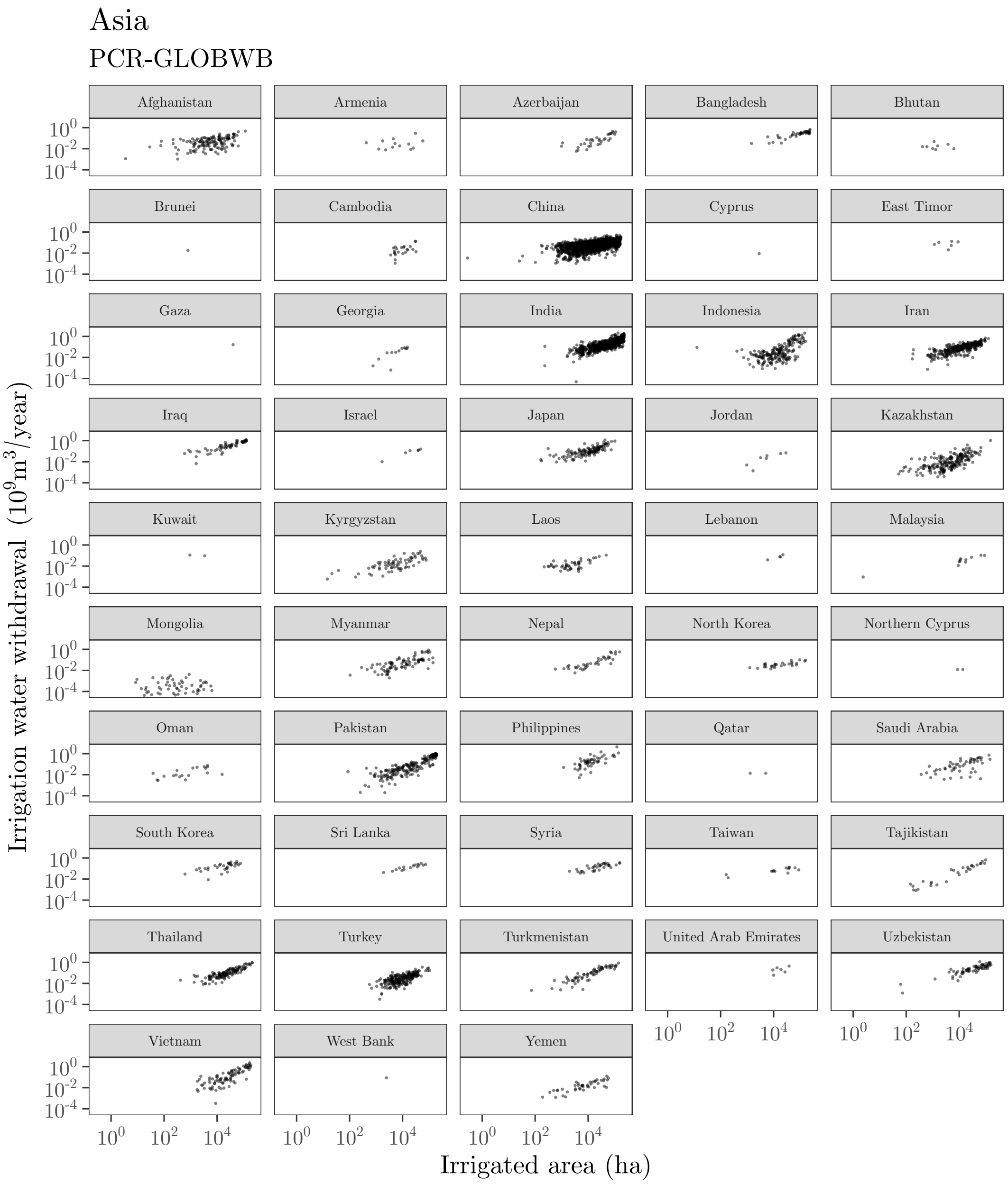

Figure S44: Irrigation water withdrawal against irrigated areas at the cell level. The data are retrieved from HYDE 3.2 [6]. Each dot is a cell.

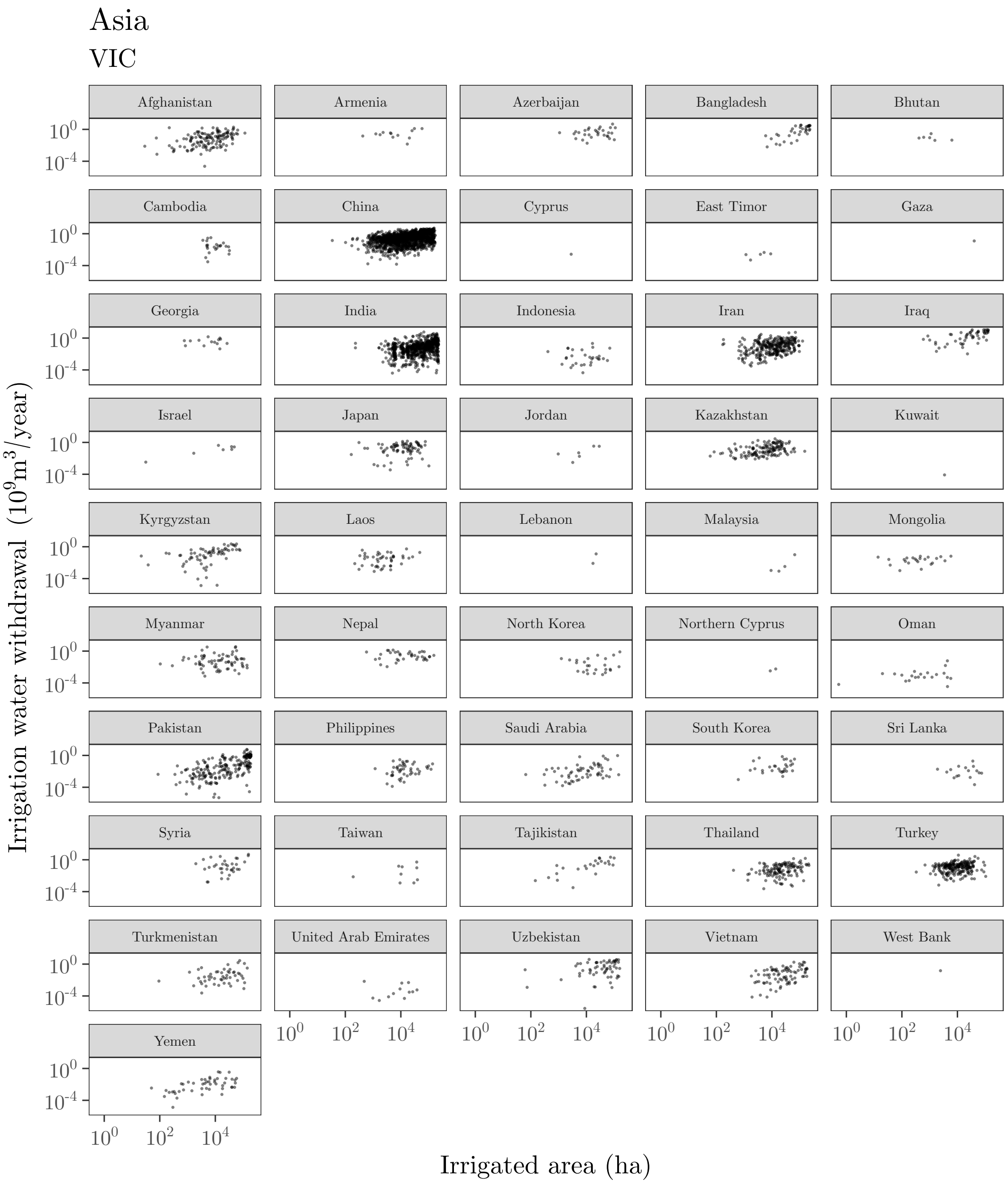

Figure S45: Irrigation water withdrawal against irrigated areas at the cell level. The data are retrieved from HYDE 3.2 [6]. Each dot is a cell.

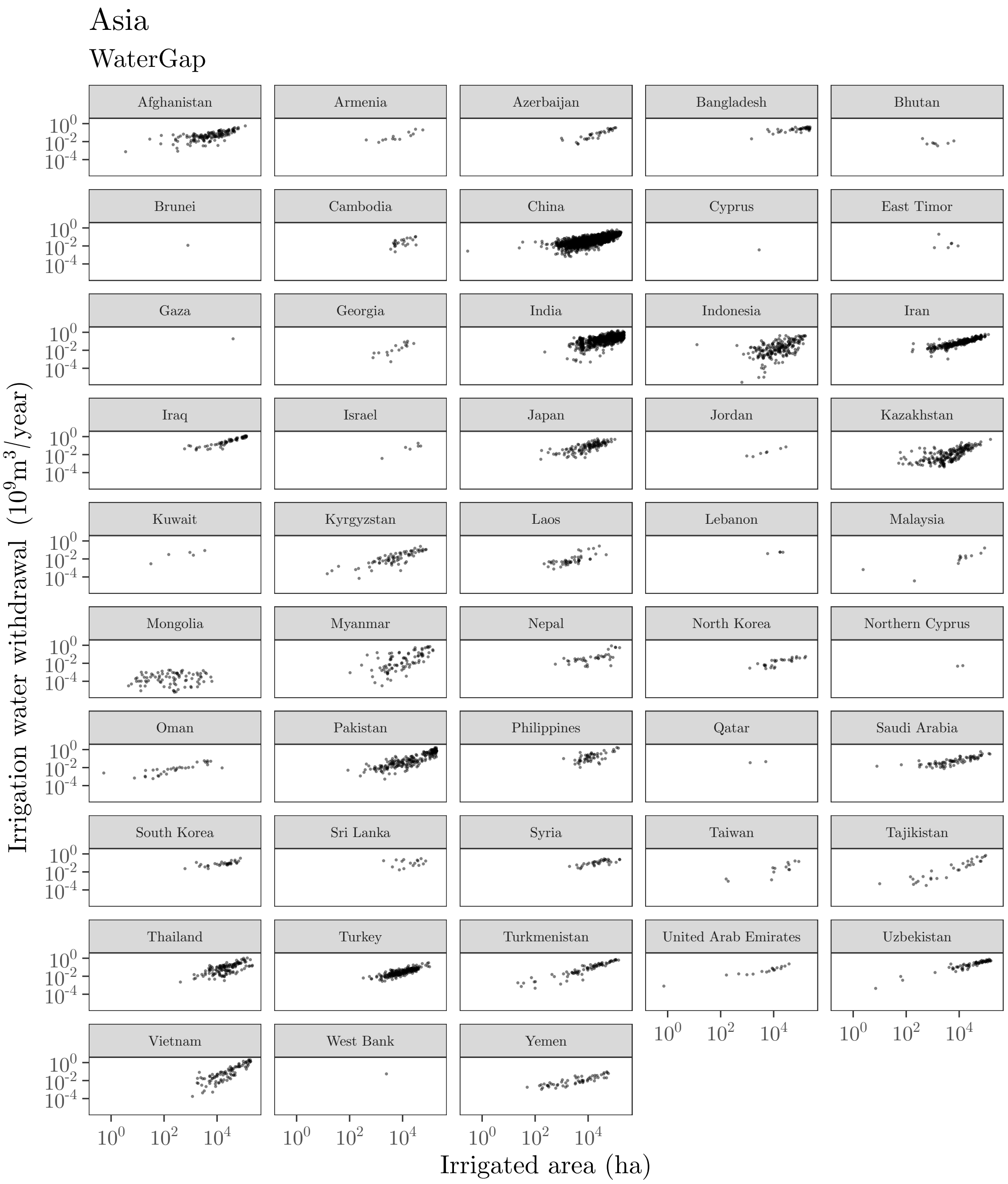

Figure S46: Irrigation water withdrawal against irrigated areas at the cell level. The data are retrieved from HYDE 3.2 [6]. Each dot is a cell.

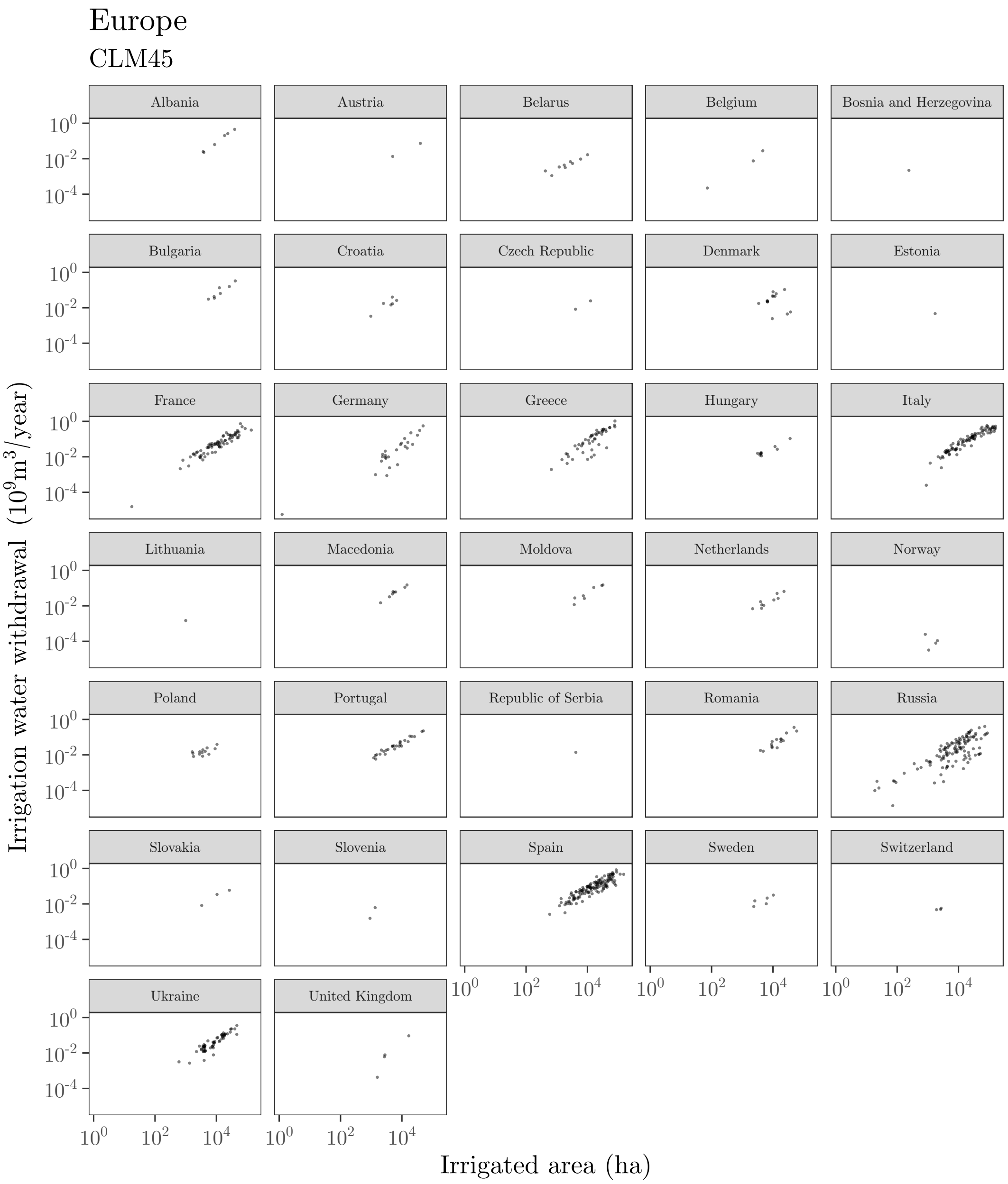

Figure S47: Irrigation water withdrawal against irrigated areas at the cell level. The data are retrieved from HYDE 3.2 [6]. Each dot is a cell.

Europe

DBHM

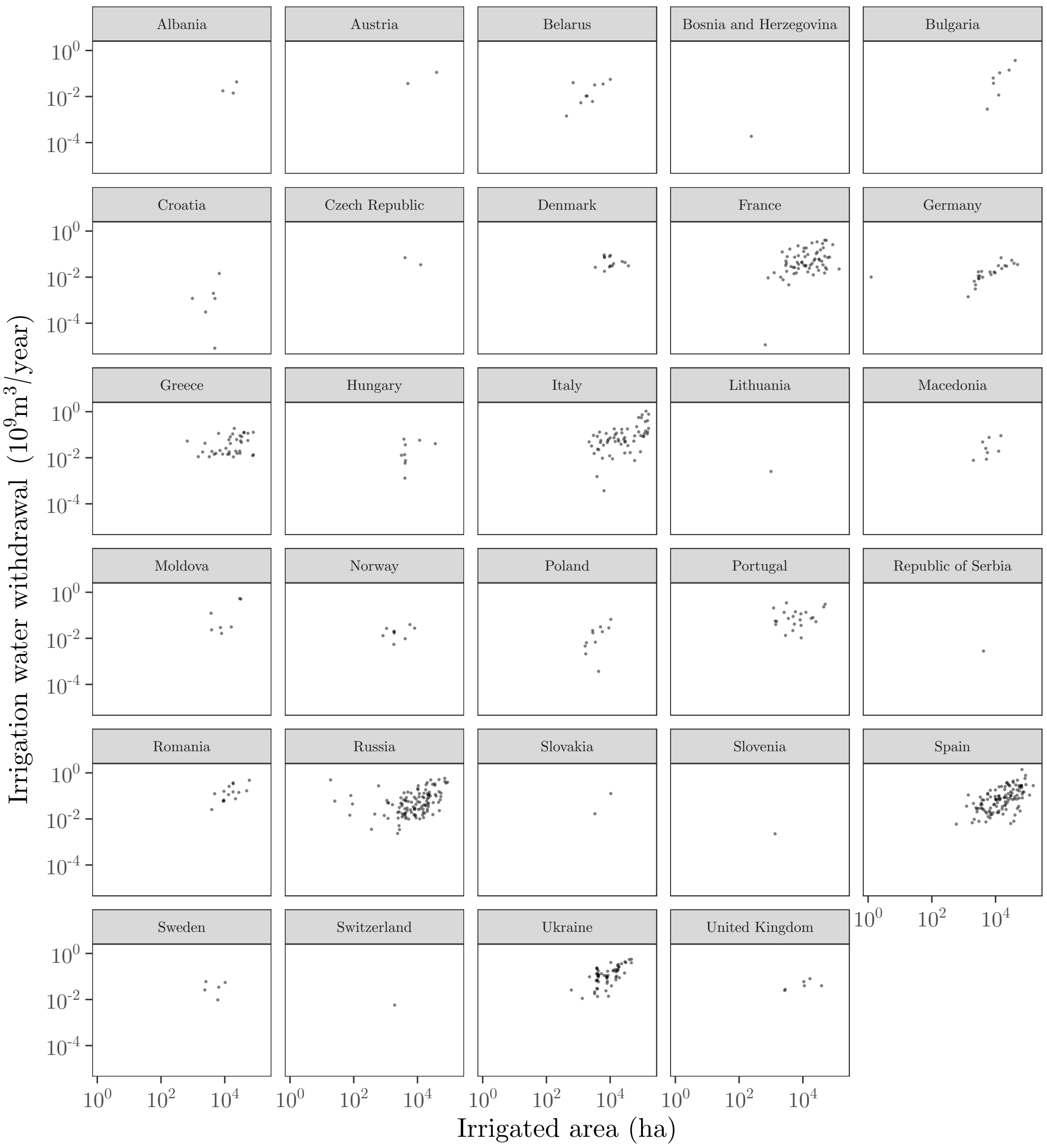

Figure S48: Irrigation water withdrawal against irrigated areas at the cell level. The data are retrieved from HYDE 3.2 [6]. Each dot is a cell.

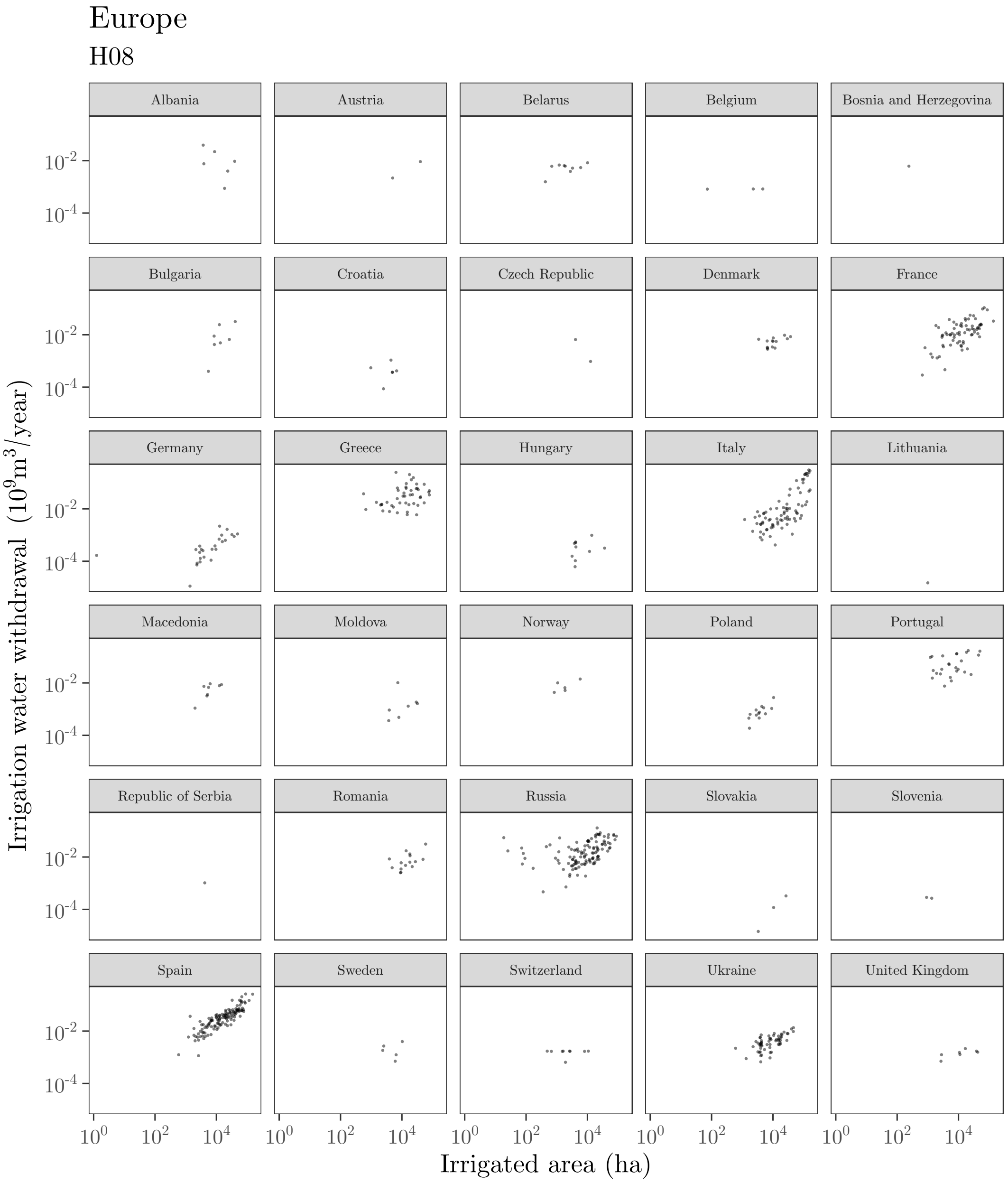

Figure S49: Irrigation water withdrawal against irrigated areas at the cell level. The data are retrieved from HYDE 3.2 [6]. Each dot is a cell.

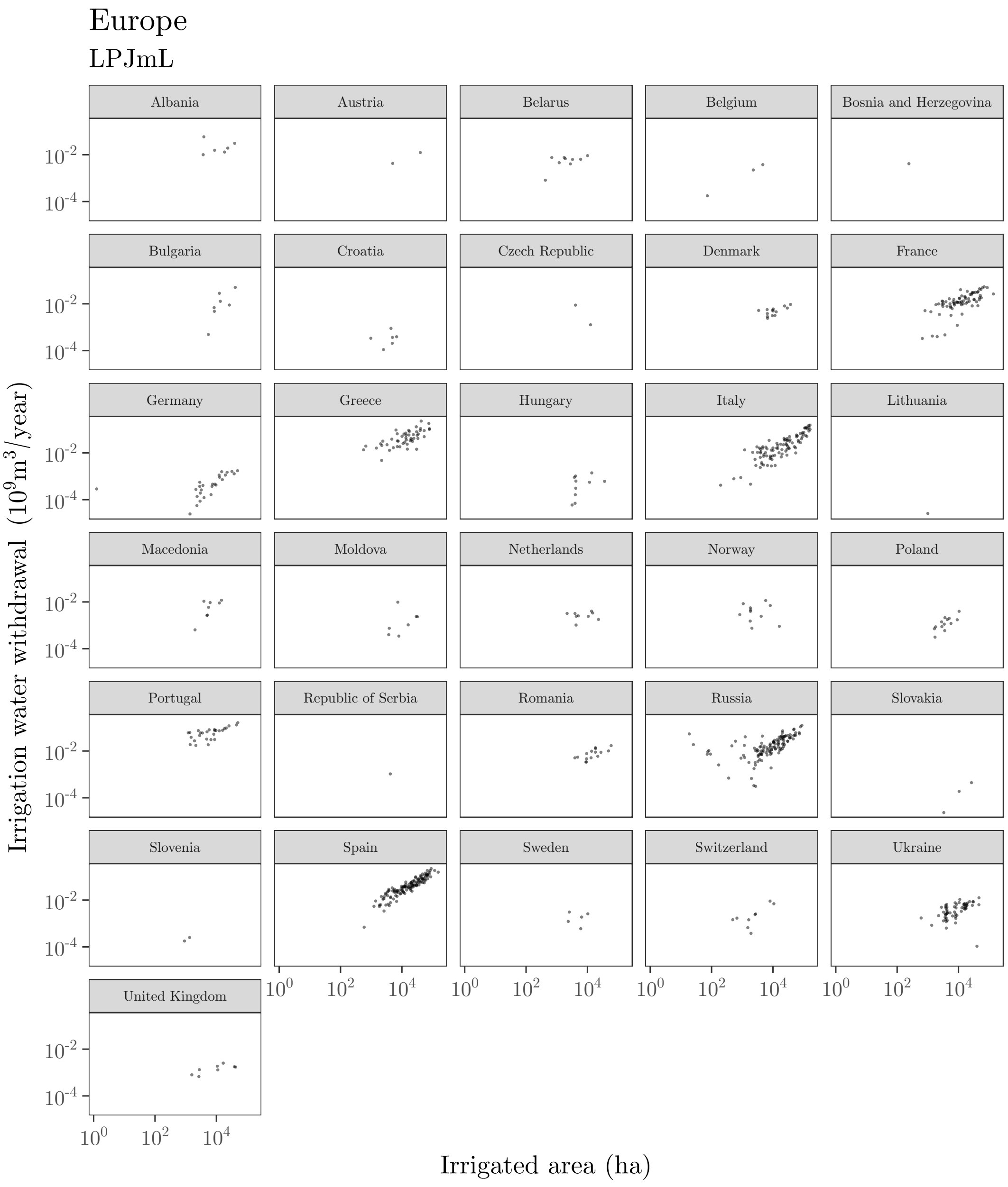

Figure S50: Irrigation water withdrawal against irrigated areas at the cell level. The data are retrieved from HYDE 3.2 [6]. Each dot is a cell.

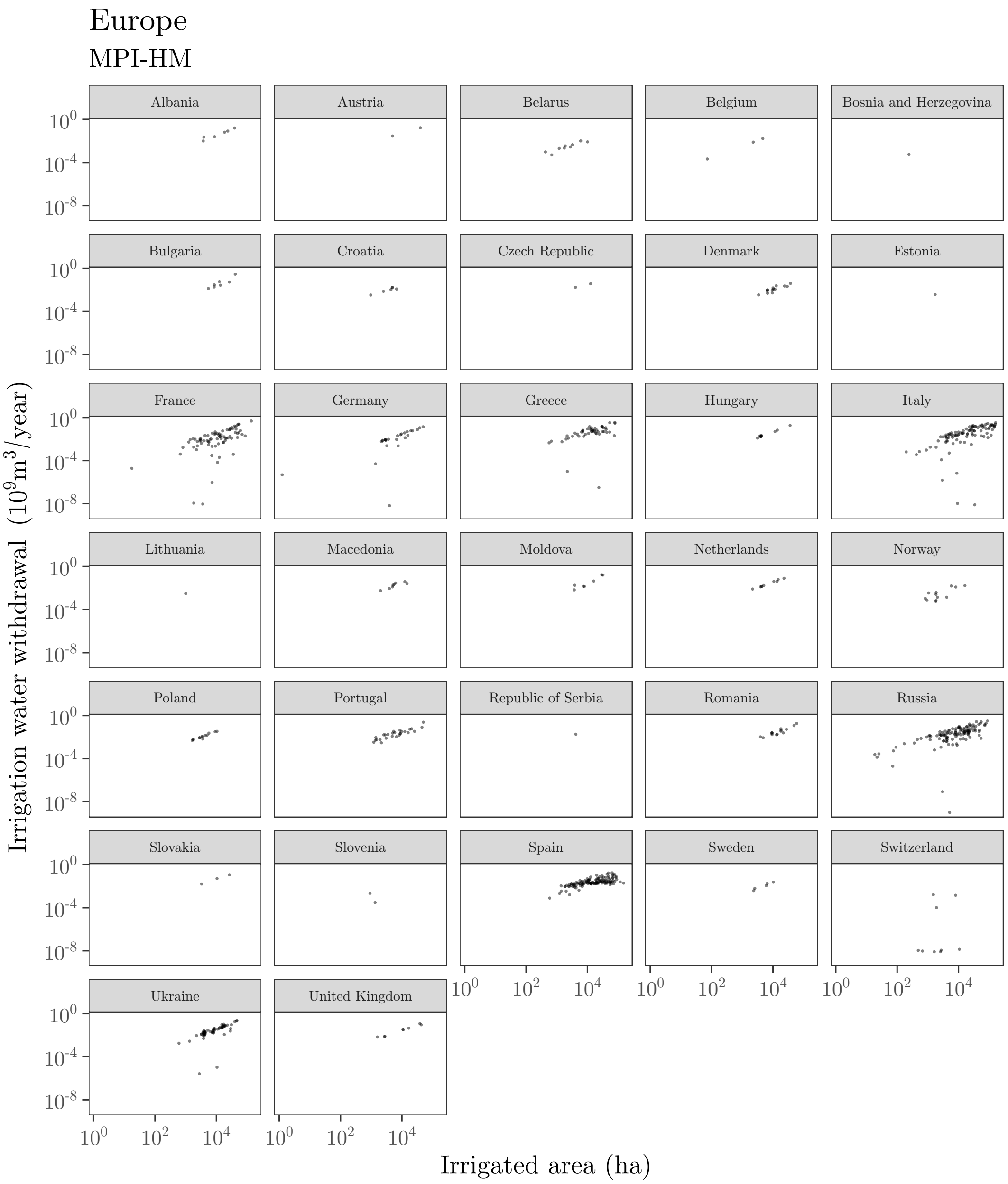

Figure S51: Irrigation water withdrawal against irrigated areas at the cell level. The data are retrieved from HYDE 3.2 [6]. Each dot is a cell.

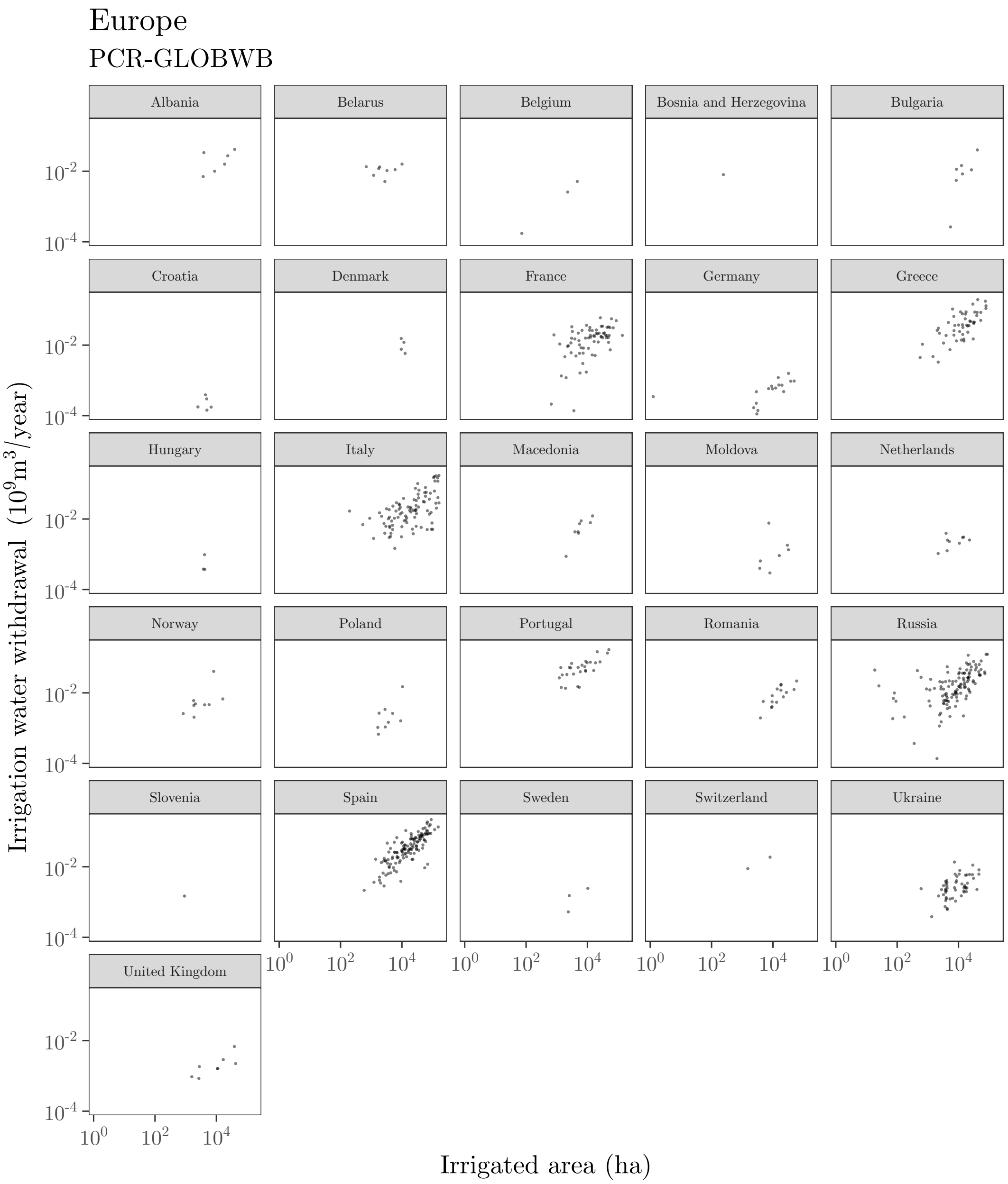

Figure S52: Irrigation water withdrawal against irrigated areas at the cell level. The data are retrieved from HYDE 3.2 [6]. Each dot is a cell.

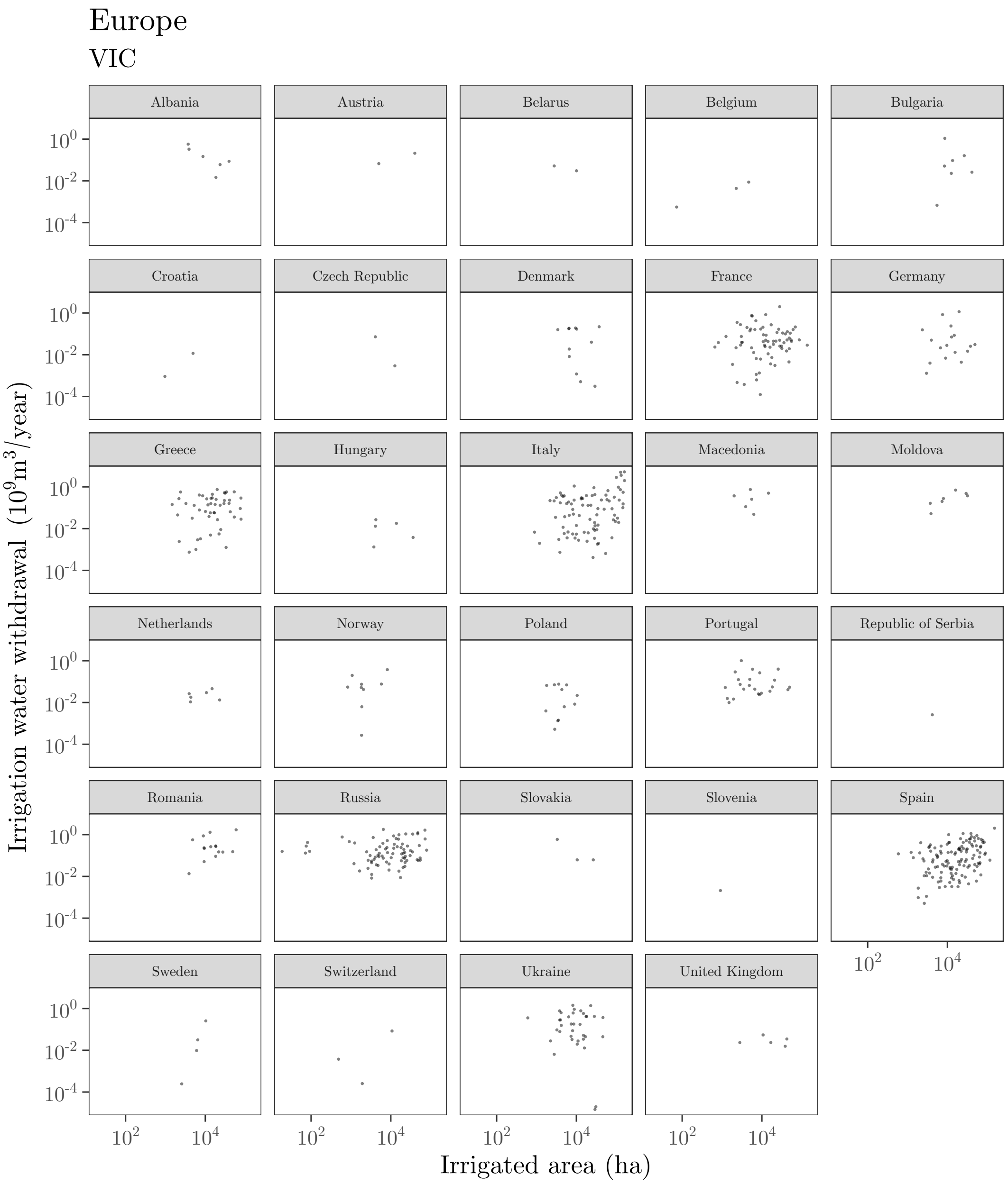

Figure S53: Irrigation water withdrawal against irrigated areas at the cell level. The data are retrieved from HYDE 3.2 [6]. Each dot is a cell.

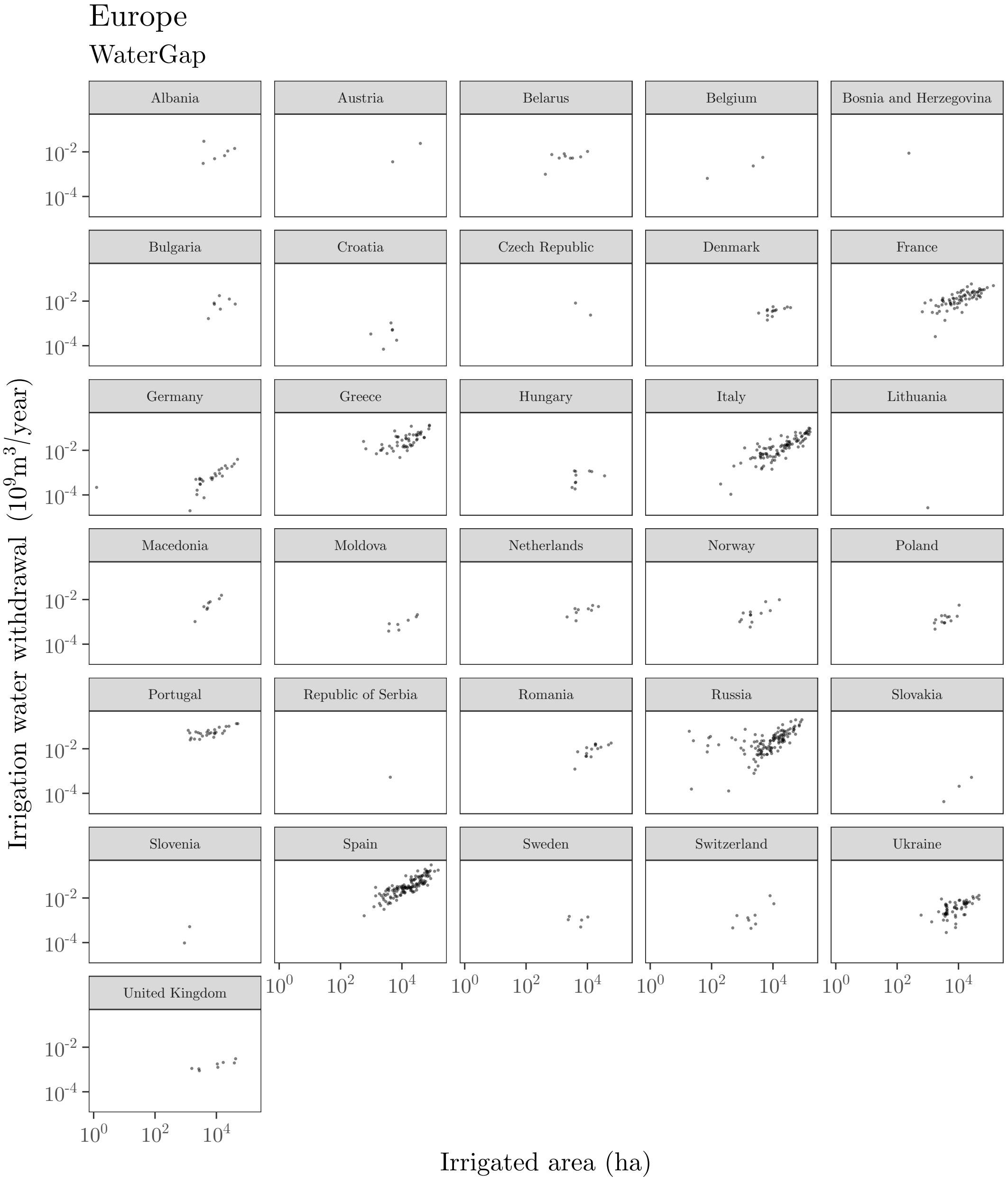

Figure S54: Irrigation water withdrawal against irrigated areas at the cell level. The data are retrieved from HYDE 3.2 [6]. Each dot is a cell.

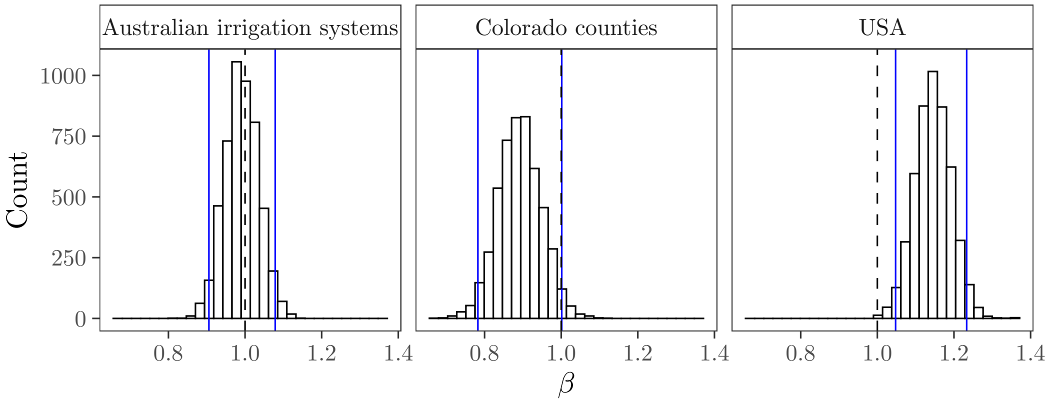

Figure S55: Distribution of β after bootstrapping ($R = 5,000$). The blue lines show the 95% confidence intervals, calculated with the bias-corrected and accelerated (bca) method [7]. The dashed vertical black line is at $\beta = 1$.

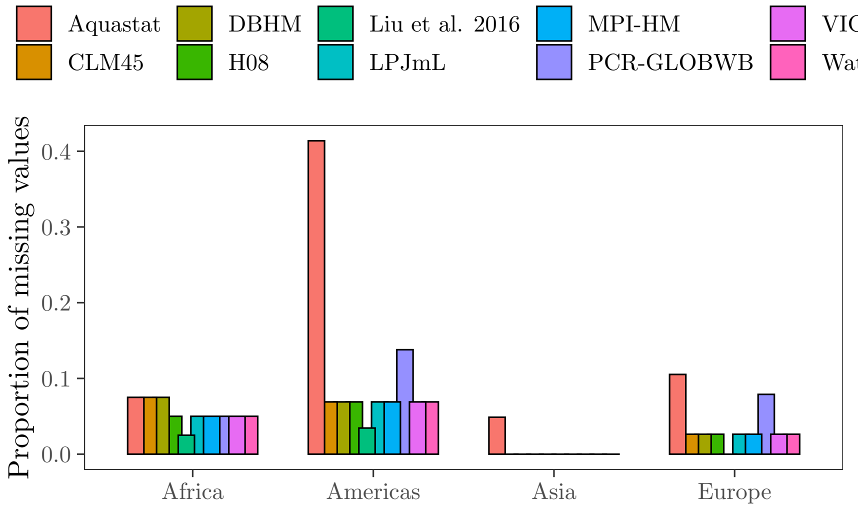

Figure S56: Proportion of countries with missing irrigation water withdrawal data.

- **Trigger**
  - Water withdrawal dataset: $X_1 \sim \mathcal{DU}(1, 6)$
    - 1: LPJmL [8]
    - 2: H08 [9, 10]
    - 3: PCR-GLOBWB [11, 12]
    - 4: WaterGap [13]
    - 5: Aquastat [14]
    - 6: Liu et al. [15]
    - 7: DBHM [16]
    - 8: VIC [17]
    - 9: MPI-HM [18]
    - 10: CLM45 [19]
  - Imputation method: $X_2 \sim \mathcal{DU}(1, 3)$
    - 1: Bayesian linear regression
    - 2: Linear regression with bootstrap
    - 3: Linear regression without model error
  - Iteration selected: $X_3 \sim \mathcal{U}(1, 40)$
  - Regression method: $X_4 \sim \mathcal{U}(1, 2)$
    - 1: Ordinary Least Squares Regression (OLS)
    - 2: Robust regression

Figure S57: Tree diagram coding the discrete probability distribution of each trigger into each uncertainty level.

$$
\boldsymbol{Q} = \begin{pmatrix}
\overbrace{\begin{matrix} X_1 & X_2 & X_3 & X_4 \\ 6 & 2 & 30 & 2 \\ 3 & 1 & 3 & 1 \\ 1 & 3 & 31 & 2 \\ 8 & 2 & 17 & 1 \\ 2 & 1 & 33 & 1 \end{matrix}}^{\boldsymbol{A}} &
\overbrace{\begin{matrix} X_1 & X_2 & X_3 & X_4 \\ 4 & 2 & 30 & 2 \\ 8 & 1 & 18 & 1 \\ 5 & 2 & 8 & 1 \\ 6 & 2 & 40 & 2 \\ 2 & 1 & 40 & 1 \end{matrix}}^{\boldsymbol{B}}
\end{pmatrix}
$$

$$
\boldsymbol{A}_B^{(1)} = \begin{pmatrix} 4 & 2 & 30 & 2 \\ 8 & 1 & 3 & 1 \\ 5 & 3 & 31 & 2 \\ 6 & 2 & 17 & 1 \\ 2 & 1 & 33 & 1 \end{pmatrix} \tag{1}
$$

$$
\boldsymbol{A}_B^{(2)} = \begin{pmatrix} 6 & 2 & 30 & 2 \\ 3 & 1 & 3 & 1 \\ 1 & 2 & 31 & 2 \\ 8 & 2 & 17 & 1 \\ 2 & 1 & 33 & 1 \end{pmatrix}
$$

$$\vdots$$

Figure S58: Example of the creation of an $\boldsymbol{A}$, $\boldsymbol{B}$ and $\boldsymbol{A}_B^{(i)}$ matrices with $k = 4$. The $\boldsymbol{Q}$ matrix has been created with Sobol' [20, 21] Quasi-Random numbers scrambled following Owen. We only show the first 5 rows of the Sobol' sequence. Note that the sequence has already been transformed in every column to the appropriate probability distribution of each trigger (see Fig. S57). The figure is based on Puy et al. [22].
